# New Meroterpenoids and α-Pyrone Derivatives Isolated from the Mangrove Endophytic Fungal Strain *Aspergillus* sp. GXNU-Y85

**DOI:** 10.3390/md22060277

**Published:** 2024-06-13

**Authors:** Chungu Wang, Fanfan Wang, Pingfang Tao, Yuanling Shao, Qing Li, Minmin Gu, Zhixin Liao, Feng Qin

**Affiliations:** 1 Department of Pharmaceutical Engineering, School of Chemistry and Chemical Engineering, Southeast University, Nanjing 211189, China; cgwang0208@163.com (C.W.); 220203070@seu.edu.cn (Y.S.); liqing12018002134@163.com (Q.L.); 220213219@seu.edu.cn (M.G.); 2 Guangxi Key Laboratory of Agricultural Resources Chemistry and Biotechnology, College of Chemistry and Food Science, Yulin Normal University, Yulin 537000, China; fanfanwang2020@163.com (F.W.); tpf005@126.com (P.T.)

**Keywords:** *Aspergillus* sp., meroterpenoids, α-pyrone derivatives, anti-proliferation activity

## Abstract

Two new meroterpenoids, aspergienynes O and P (**1** and **2**), one new natural compound, aspergienyne Q (**3**), and a new α-pyrone derivative named 3-(4-methoxy-2-oxo-2H-pyran-6-yl)butanoic acid (**4**) were isolated from the mangrove endophytic fungal strain *Aspergillus* sp. GXNU-Y85, along with five known compounds (**5**–**9**). The absolute configurations of those new isolates were confirmed through extensive analysis using spectroscopic data (HRESIMS, NMR, and ECD). The pharmacological study of the anti-proliferation activity indicated that isolates **5** and **9** displayed moderate inhibitory effects against HeLa and A549 cells, with the IC_50_ values ranging from 16.6 to 45.4 μM.

## 1. Introduction

Endophytic fungi, as a type of fungi, persist for an extended period in the healthy tissues and organs of plants, which can be regarded as a novel resource of microorganisms with high exploitation value and an important source of natural active substances [1,2]. Therefore, the development and utilization of endophytic fungi can alleviate the shortage of resources and the destruction of ecological balance caused by extracting and separating a large number of beneficial bioactive products from plants, and also help protect rare and endangered plant resources [3]. Some secondary metabolites isolated from endophytic fungi have also drawn attention owing to their superior biological activities. Consequently, these secondary metabolites might be used as promising lead compounds for drug discovery [4].

**Figure 1:**
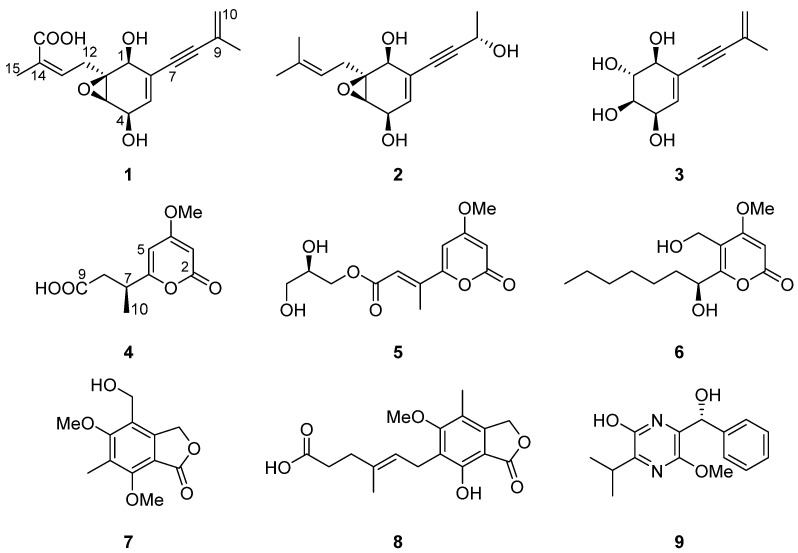
The chemical structures **1**–**9**.

In a continuous endeavour to identify the bioactive constituents derived from mangrove endophytic fungi [5,6], we isolated two new meroterpenoids, aspergienynes O and P (**1** and **2**), one new natural compound, aspergienyne Q (**3**), and a new α-pyrone derivative termed 3-(4-methoxy-2-oxo-2H-pyran-6-yl)butanoic acid (**4**), together with five previously reported compounds (**5**–**9**), from the secondary metabolites of *Aspergillus* sp. GXNU-Y85 derived from the fruits of the mangrove plant *Kandelia candel* in this study. Five kinds of human cancer cell lines were selected to detect the anti-proliferation activity of these isolates, and the separation and structure clarification process of **1**–**9** were described in detail (Figure 1).

## 2. Results and Discussion

### 2.1. Process of Structural Characterization of Isolates

**Figure 2:**
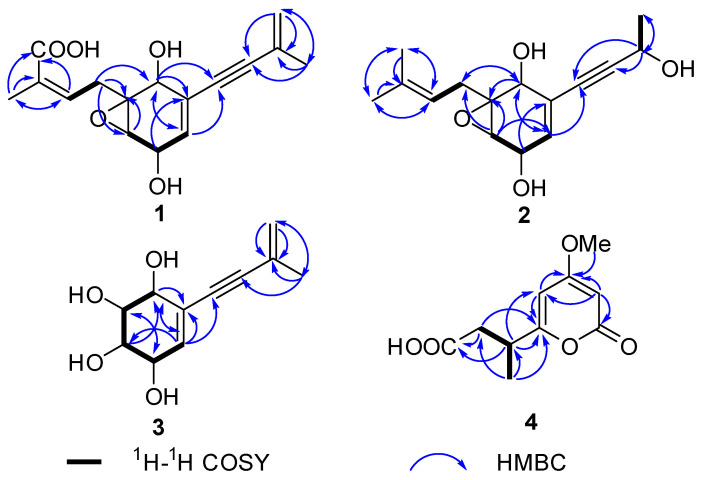
Key HMBC and ^1^H-^1^H COSY correlations of compounds **1**–**4**.

Aspergienyne O (**1**) was acquired as a yellowish oil. Its HR-ESI-MS peak at *m*/*z* 313.1048 ([M + Na]^+^, calcd for 313.1046) and the ^13^C NMR data (Table 1) suggested a molecular formula of C_16_H_18_O_5_ with eight degrees of unsaturation. The ^1^H NMR and HSQC data of **1** (Table 1) exhibited four olefinic groups [*δ*_H_ 6.72 (m), 5.77 (dd, *J* = 5.0, 1.9 Hz), 5.28 (m) and 5.25 (m)], a methylene [*δ*_H_ 3.03 (dd, *J* = 15.1, 8.8 Hz), and 2.54 (dd, *J* = 15.1, 6.9 Hz)], three oxymethines [*δ*_H_ 4.46 (dd, *J* = 5.0, 2.5 Hz), 4.19 (t, *J* = 1.9 Hz), and 3.34 (t, *J* = 2.5 Hz)], and two methyls [*δ*_H_ 1.90 (s) and 1.87 (s)]. The ^13^C NMR and HSQC data of **1** displayed sixteen carbons, such as a carbonyl carbon (*δ*_C_ 171.7), six sp^2^ carbons (*δ*_C_ 141.1, 134.3, 132.6, 128.4, 124.3, and 122.3), two sp carbons (*δ*_C_ 91.7 and 87.5), three sp^3^ methine carbons (*δ*_C_ 68.1, 66.1, and 60.3), one sp^3^ nonprotonated carbon (*δ*_C_ 62.5), one sp^3^ methylene group (*δ*_C_ 32.5), together with two methyl carbons (*δ*_C_ 23.5 and 12.9). The NMR data (Table 1) of **1** were highly similar to those of the previously reported biscognienyne D [7], except that the methyl group was replaced by the carboxyl group of **1**. The HMBC signals from H-15/H-13 to C-16 (*δ*_C_ 171.7) supported this speculation (Figure 2). Comprehensive analysis of its 2D NMR signals not only confirmed the above hypothesis but also verified the two-dimensional structure shown in Figure 1.

**Figure 3:**
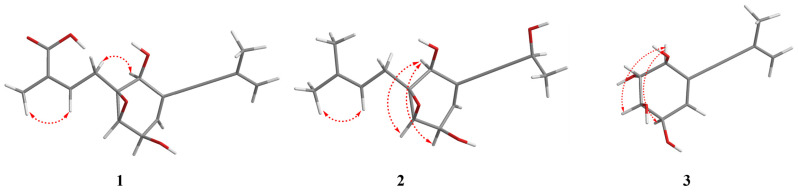
Key NOESY correlations for **1**–**3**.

**Figure 4:**
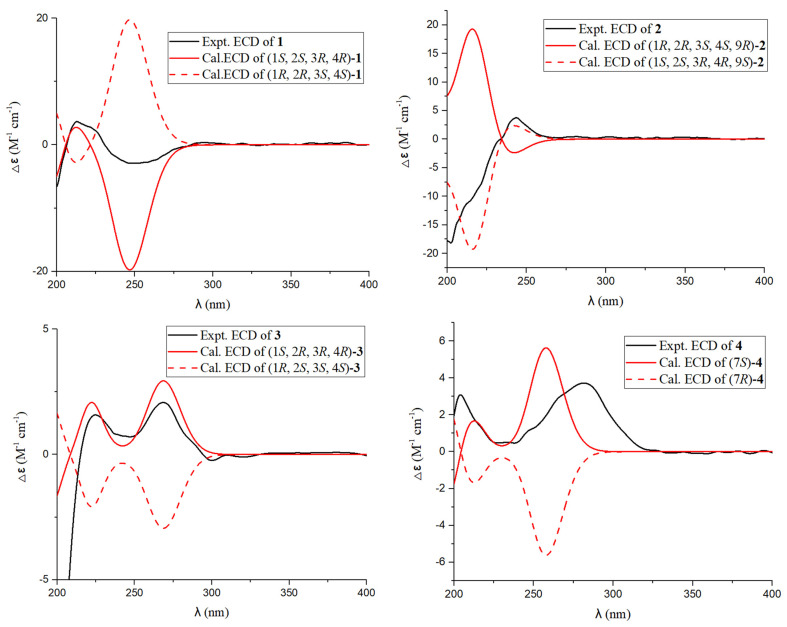
Calculated and experimental ECD spectra of **1**–**4**.

In the NOESY spectrum of **1** (Figure 3), there is a diagnostic association between the olefinic proton [*δ*_H_ 6.72 (m, H-13)] and the terminal methyl proton [*δ*_H_ 1.87 (s, H_3_-15)], and thus the geometry of the Δ^13,14^ double bond was defined as *Z*. Furthermore, the NOESY signals of H-1/H-12β suggested that H-1 and H-12β were β-oriented. Meanwhile, the small value of *J*_H-3/H-4_ (2.5 Hz) demonstrated a *cis*-orientation of these protons. The final configuration of **1** was elucidated as (1*S*,2*S*,3*R*,4*R*)-**1** by means of comparing its experimental ECD spectrum to those calculated (Figure 4).

**Figure 5:**
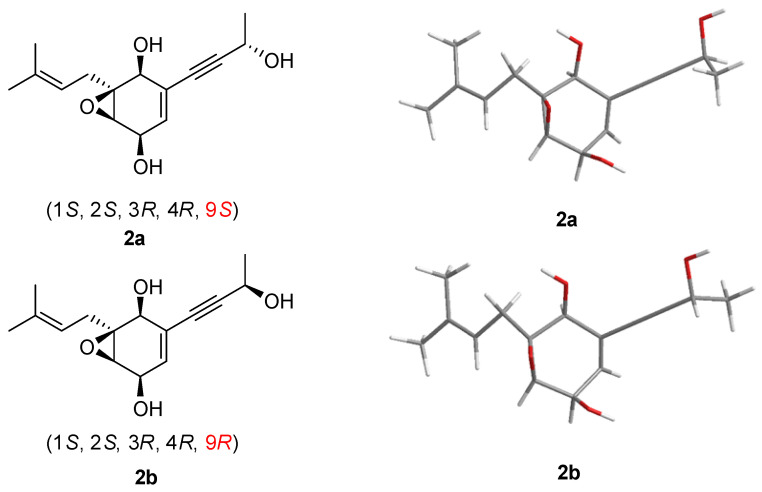
Conformations of low-energy conformers of structures **2a** and **2b** in MeOH.

**Figure 6:**
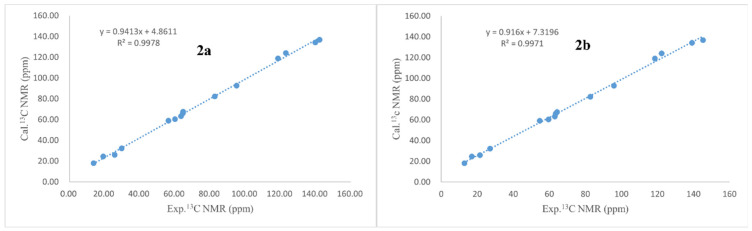
Regression analyses of experimental versus calculated ^13^C NMR chemical shifts of model compounds **2a** and **2b**.

Aspergienyne P (**2**) was isolated as a yellowish oil. The analysis of the ion peak discovered at *m*/*z* 287.1255 ([M + Na]^+^, calcd for 287.1259) in the HR-ESI-MS report combined with its ^13^C NMR data gained the molecular formula C_15_H_20_O_4_. Comprehensive analysis of 1D NMR information (Table 1) of **2** proposed that **2** and 1 are very similar in structure, except for the replacement of 16-COOH and the Δ^9,10^ double bond in **1** by a methyl group and a hydroxyl group in **2**, respectively, which was determined via the crucial HMBC signals from H-13/H-15 to C-16 (*δ*_C_ 18.1), H-16 (*δ*_H_ 1.69) to C-13/C-14/C-15, H-9 (*δ*_H_ 4.57) to C-7/C-8, H-10 to C-9 (*δ*_C_ 59.0), and the significant ^1^H-^1^H COSY signals (Figure 2) of H-9/H-10. The NOESY signals (Figure 3) of H-1/H-3 and H-1/H-4 suggested that H-1, H-3, and H-4 were on the same side. Consequently, (1S*, 2S*, 3R*, 4R*)-**2** was tentatively established. The ^13^C NMR data of two potential isomers (**2a** and **2b**) (Figure 5) were calculated according to the GIAO method to determine the complete relative configuration of **2**. Due to the good correlation coefficient (R^2^ = 0.9978) (Figure 6) and the high results of the DP4+ probability analysis (100%) (Table S2) between the calculated and experimental ^13^C NMR chemical shifts, the (1S*,2S*,3R*,4R*,9S*)-**2** was recommended. The absolute stereochemistry of **2** was determined as 1*S*, 2*S*, 3*R*, 4*R**,*** and 9*S* due to the coincidence of the calculated and experimental ECD spectral curves (Figure 4).

The C_11_H_14_O_4_ of aspergienyne Q (**3**) was determined on the basis of HR-ESI-MS analysis, which exhibited a [M +Na]^+^ ion at *m*/*z* 233.0785 (calcd for 233.0784). The NMR data (Table 2) of **3** was compared in detail with that of (1*R*,2*R*,3*R*,4*S*)-5-(3-Methylbut-3-en-1-yn-1-yl)-cyclohex-5-ene-1,2,3,4-tetraol [8], and combined with the HR-ESI-MS of **3**, and the structure of compound **3** was determined. Moreover, the absolute structure of **3** was further confirmed by 2D NMR and ECD spectroscopy (Figure 2, Figure 3 and Figure 4). Thus, as shown in Figure 1, **3** is reported for the first time in this paper as a new natural product.

The molecular formula of 3-(4-methoxy-2-oxo-2H-pyran-6-yl)butanoic acid (**4**) was defined as C_10_H_12_O_5_ according to the HR-ESI-MS ion peak discovered at *m*/*z* 213.0765 ([M + H]^+^, calcd for 213.0763), implying five degrees of unsaturation. The ^1^H NMR spectrum displayed the presence of two olefins [*δ*_H_ 6.04 (d, *J* = 2.2 Hz) and 5.54 (d, *J* = 2.2 Hz)], a methylene [*δ*_H_ 2.58 (dd, *J* = 14.6, 7.4 Hz) and 2.40 (dd, *J* = 14.6, 7.5 Hz)], one methine proton [*δ*_H_ 3.10 (m)], a methyl proton [*δ*_H_ 1.26 (d, *J* = 7.0 Hz)], and one methoxy [*δ*_H_ 3.85 (s)]. Subsequently, the carbon resonances of one methine group (*δ*_C_ 36.4), one methylene (*δ*_C_ 40.7), one methyl (*δ*_C_18.3), and one methoxy (*δ*_C_ 56.9) were observed in its ^13^C NMR spectrum, along with two carbonyl carbons (*δ*_C_ 176.0 and 167.4) and four olefin carbons (*δ*_C_ 173.0, 169.2, 100.7, and 88.4), as supported by its HSQC spectrum. The NMR data of **4** closely resembled those of pestalotiopyrone N [9], except for a Δ^7,8^ double bond in pestalotiopyrone N being replaced by one methine and one methylene present at C-7 and C-8 in **4**, which was proved on the basis of the key HMBC signals from H-7 to C-5/C-6/C-9, H-8 to C-6/C-9 and H-10 to C-6/C-7, and the key ^1^H-^1^H COSY signals (Figure 2) of H-7/H-8 and H-7/H-10. Finally, the absolute configuration of (7*S*)-**4** was elucidated via ECD calculations (Figure 4).

In addition to these four new isolates, five previously reported isolates were acquired, which were identified on the basis of comparing their spectral data with the reported data as pestalotiopyrone I (**5**) [10], ficipyrone A (**6**) [11], 4-(hydroxymethyl)-5,7-dimethoxy-6-methylisobenzofuran-1(3H)-one (**7**) [12], mycophenolic acid (**8**) [13], and terezine A (**9**) [14].

### 2.2. Results of Antiproliferative Activity

The MTT method was used to evaluate the anti-proliferative activity of all isolates on five human cancer cell lines. Among them, **9** displayed moderate anti-proliferation activity on HeLa cancer cells (IC_50_ =16.6 μM); moreover, **5** displayed anti-proliferation activity on the HeLa and A549 cancer cell lines (IC_50_ = 29.3 μM and IC_50_ = 45.4 μM, respectively). The other isolates displayed no notable anti-proliferation activity on five cancer cell lines (IC_50_ > 50 μM). The positive control was etoposide (IC_50_: 15.7 μM for HeLa cells, 8.2 μM for A549 cells).

## 3. Materials and Methods

### 3.1. General Experimental Procedures

The NMR data were measured by Bruker 400 MHz and 600 MHz instruments (Bruker, Bremen, Germany). The HR-ESI-MS reports and the Optical rotations were collected using an LC-MS spectrometer (Agilent 6545 Q-TOF) and a JASCO P-2000 polarimeter (Jasco, Tokyo, Japan), respectively. The other instruments and materials employed in the experiments were the same as those in our previous reports [15].

### 3.2. Fungal Material

According to the sequence and morphology of the internal transcriptional spacer (ITS) of the strain, the fungal strain collected from fresh fruits of the mangrove plant *Kandelia candel* in the Beihai was identified and designated as GXNU-Y85. Subsequently, we obtained its registration number OR999402, as we submitted the ITSrDNA of GXNU-Y85 to GenBank.

### 3.3. Fermentation, Extraction, and Isolation

This target strain was fermented for 28 days at 25 °C, where the medium consisted of 80 mL H_2_O (H_2_O with 0.5% sea salt) and 80 g of rice. Mycelium was collected and soaked in methanol (3 × 10 L) for 2 days to collect the crude extract (21.4 g), which was subsequently extracted 3 times using ethyl acetate. The extract (11.1 g) was isolated via a silica gel column, and six fractions (Fr. 1-Fr. 6) were obtained using a ratio of PE-EtOAc solvent (50:1 to 1:1, *v*/*v*). Fr. 5 (2.54 g) was further isolated through a Sephadex LH-20 column (100% methanol) to produce seven fractions (Fr. 5.1 to Fr. 5.7). Isolates **3** (5.6 mg), **8** (7.1 mg), and **5** (3.9 mg) were separated from Fr. 5.2 (0.84 g) based on semipreparative HPLC (50% MeOH–H_2_O; 7 mL/min; YMC-column, 4.6 mm I.D. × 250 mm, S-5 μm, 12 nm). Isolates **2** (4.6 mg), **7** (6.3 mg), **1** (7.6 mg), and **6** (4.8 mg) were acquired from Fr. 5.4 (0.92 g) on the basis of semipreparative HPLC (MeCN–H_2_O *v*/*v*, 40:60; 7 mL/min; YMC-column, 4.6 mm I.D. × 250 mm, S-5 μm, 12 nm). Fr. 5.7 (0.71 g) was further separated according to semipreparative HPLC (MeCN–H_2_O *v*/*v*, 40:60; 7 mL/min; YMC-column, 4.6 mm I.D. × 250 mm, S-5 μm, 12 nm) to yield **4** (5.2 mg) and **9** (6.1 mg).

#### 3.3.1. Aspergienyne O (**1**)

Yellowish oil; ${\left[\alpha \right]}_{D}^{22}$ −13.75 (*c* 0.37, MeOH); UV (MeOH) *λ*_max_ (log ε) 259.5 (3.25) nm; IR (KBr) *ν*_max_ 3357, 2977, 2930, 2195, 1671, 1448, and 1039 cm^−1^; HR-ESI-MS *m*/*z* [M + Na]^+^ (313.1048); ECD (MeOH) λmax (Δε) 213.50 (+3.769), 247.74 (−2.965) nm; NMR data (in CD_3_OD) at Table 1.

#### 3.3.2. Aspergienyne P (**2**)

Yellowish oil; ${\left[\alpha \right]}_{D}^{22}$ −41.26 (*c* 0.30, MeOH); UV (MeOH) *λ*_max_ (log ε) 258.1 (3.15) nm; IR (KBr) *ν*_max_ 3421, 1719, 1609, 1430, 1384, 1247, 830, and 787 cm^−1^; HR-ESI-MS *m*/*z* [M + Na]^+^ (287.1255); ECD (MeOH) λmax (Δε) 202.50 (−18.463), 243.09 (+4.011) nm; NMR data (in CD_3_OD) at Table 1.

#### 3.3.3. Aspergienyne Q (**3**)

Yellowish oil; ${\left[\alpha \right]}_{D}^{22}$ +16.89 (*c* 0.69, MeOH); UV (MeOH) *λ*_max_ (log ε) 259.2 (3.02) nm; IR (KBr) *ν*_max_ 3428, 2955, 2870, 1656, 1459, 1382, 1370, 1038, 967, and 834 cm^−1^; HR-ESI-MS *m*/*z* [M + Na]^+^ (233.0785); ECD (MeOH) λmax (Δε) 224.76 (+1.570), 268.54 (+2.080) nm; NMR data (in CD_3_OD) at Table 2.

#### 3.3.4. 3-(4-Methoxy-2-oxo-2H-pyran-6-yl)butanoic Acid (**4**)

Yellowish oil; ${\left[\alpha \right]}_{D}^{22}$ −15.03 (*c* 0.28, MeOH); UV (MeOH) *λ*_max_ (log ε) 212.7 (3.08), 264.4 (3.31) nm; IR (KBr) *ν*_max_ 3411, 2933, 1638, 1464, 1371, 1034, and 886 cm^−1^; HR-ESI-MS *m*/*z* [M + H]^+^ (213.0765); ECD (MeOH) λmax (Δε) 203.65 (+3.078), 281.71 (+3.744) nm; NMR data (in CD_3_OD) at Table 2.

### 3.4. ECD and NMR Calculations

The absolute stereochemistry of the new isolates was defined through the ECD and NMR calculations described in previous reports [16,17]. The Supporting Information provides a detailed description of the process.

### 3.5. Anti-Proliferative Activity Test

The anti-proliferative activity of **1**–**9** against five cancer cell lines (A549, MCF-7, Hela, 5-8F, and T24 cells) was evaluated by MTT assay as previously reported [18]. The positive control was etoposide.

## 4. Conclusions

In the present study, three new compounds (**1**, **2**, and **4**), one new natural compound (**3**), and five previously reported isolates (**5**–**9**) were acquired from the fungal strain *Aspergillus* sp. GXNU-Y85 by various chromatographic techniques. Extensive spectroscopic data (HRESIMS, NMR, and ECD) and quantum chemical calculations were used to determine the structures of these new compounds. Biological activity studies revealed that compounds **5** and **9** showed moderate anti-proliferative activity against HeLa and A549 cells (IC_50_ = 16.6–45.4 μM).

## Figures and Tables

**Table 1:** ^1^H and ^13^C NMR Data for **1** and **2** in CD_3_OD.

No	1 ^a^		2 ^b^	
	*δ*_C_, Type	*δ*_H_ (*J* in Hz)	*δ*_C_, Type	*δ*_H_ (*J* in Hz)
1	68.1, CH	4.19, t (1.9)	67.6, CH	4.15, t (2.0)
2	62.5, C		63.2, C	
3	60.3, CH	3.34, t (2.5)	60.3, CH	3.3, s
4	66.1, CH	4.46, dd (5.0, 2.5)	66.1, CH	4.41, dd (4.9, 2.0)
5	134.3, CH	5.77, dd (5.0, 1.9)	134.2, CH	5.74, dd (4.9, 2.3)
6	124.3, C		124.1, C	
7	87.5, C		82.3, C	
8	91.7, C		92.7, C	
9	128.4, C		59.0, CH	4.57, q (6.6)
10	122.3, CH_2_	5.28, m, 5.25, m	24.6, CH_3_	1.41, d (6.6)
11	23.5, CH_3_	1.90, s		
12	32.5, CH_2_	3.03, dd (15.1, 8.8) 2.54, dd (15.1, 6.9)	32.2, CH_2_	2.94, dd (14.6, 9.0) 2.14, dd (14.6, 6.2)
13	136.2, CH	6.72, m	119.0, CH	5.13, m
14	132.6, C		136.7, C	
15	12.9, CH_3_	1.87, s	26.0, CH_3_	1.73, s
16	171.7, C		18.1, CH_3_	1.69, s

^a 1^H NMR at 600 MHz, ^13^C NMR at 150 MHz; ^b 1^H NMR at 400 MHz, ^13^C NMR at 100 MHz.

**Table 2:** ^1^H (400 MHz) and ^13^C (100 MHz) NMR Data for **3** and **4** in CD_3_OD.

No	3		No	4	
	*δ*_C_, Type	*δ*_H_ (*J* in Hz)		*δ*_C_, Type	*δ*_H_ (*J* in Hz)
1	74.4, CH	3.86, dd (7.2, 0.8)	2	167.4, C	
2	73.4, CH	3.71, dd (9.9, 7.2)	3	88.4, CH	5.54 d (2.2)
3	72.1, CH	3.48, dd (9.9, 4.3)	4	173.9, C	
4	67.6, CH	4.23, dd (5.2, 4.3)	5	100.7, CH	6.04 d (2.2)
5	134.1, CH	6.06, dd (5.2, 1.7)	6	169.2, C	
6	128.3, C		7	36.4, CH	3.10 m
7	87.5, C		8	40.7, CH_2_	2.58 dd (14.6, 7.4), 2.40 dd (14.6, 7.5)
8	92.5, C		9	176.0, C	
9	128.3, C		10	18.3, CH_3_	1.26 d (7.0)
10	122.5, CH_2_	5.30, m; 5.28, m	4-OMe	56.9, CH_3_	3.85 s
11	23.5, CH_3_	1.91, m			

## Data Availability

The authors declare that all data of this study are available within the article and its Supplementary Materials file or from the corresponding authors upon request.

## Supplementary Materials

The following supporting information can be downloaded at: https://www.mdpi.com/article/10.3390/md22060277/s1, NMR, IR, UR and HRESIMS spectra of **1**–**4**. NMR spectra of **5**–**9**.
