# Supplementary material for: New Meroterpenoids and α-Pyrone Derivatives Isolated from the Mangrove Endophytic Fungal Strain Aspergillus sp. GXNU-Y85
Source: Mar Drugs. 2024 Jun 13;22(6):277. doi: 10.3390/md22060277 (PMC11205070; doi:10.3390/md22060277)
Supplement: Supplementary file 1 [file marinedrugs-22-00277-s001.zip › marinedrugs-3036028-supplementary.pdf]

Supplementary materials

New Meroterpenoids and α-pyrone Derivatives Isolated from the Mangrove Endophytic Fungal Strain *Aspergillus* sp. 3 GXNU-Y85

Chungu Wang [1 †], Fanfan Wang [2, †], Pingfang Tao [2], Yuanling Shao [1], Qing Li [1], Minmin Gu [1], Zhixin Liao [1, *], Feng Qin [2, *]

[1] Department of Pharmaceutical Engineering, School of Chemistry and Chemical Engineering, Southeast University, Nanjing 211189, PR China.

[2] Guangxi Key Laboratory of Agricultural Resources Chemistry and Biotechnology, College of Chemistry and Food Science, Yulin Normal University, Yulin 537000, P. R. China.

[*] Corresponding author.

E-mail address: zxliao@seu.edu.cn (Zhixin Liao), qinf1114@163.com (Feng Qin)

[†]These authors contributed equally to this work.

Contents

Figure S1 ^{1}H NMR spectrum of compound **1** (600 MHz, CD_3OD)........................... 4

Figure S2 ^{13}C NMR spectrum of compound **1** (150 MHz, CD_3OD) 4

Figure S3 COSY spectrum of compound **1** 5

Figure S4 HSQC spectrum of compound **1** 5

Figure S5 HMBC spectrum of compound **1** 6

Figure S6 NOESY spectrum of compound **1**.. 6

Figure S7 (+) HRESIMS Spectrum of compound **1**.. 7

Figure S8 IR spectrum of compound **1**.. 7

Figure S9 UV Spectrum of compound **1** 8

Figure S10 ^{1}H NMR spectrum of compound **2** (400 MHz, CD_3OD)......................... 8

Figure S11 ^{13}C NMR spectrum of compound **2** (100 MHz, CD_3OD)......................... 9

Figure S12 COSY spectrum of compound **2**.. 9

Figure S13 HSQC spectrum of compound **2** 10

Figure S14 HMBC spectrum of compound **2** 10

Figure S15 NOESY spectrum of compound **2**..11

Figure S16 (+) HRESIMS Spectrum of compound **2**..11

Figure S17 IR spectrum of compound **2**.. 12

Figure S18 UV Spectrum of compound **2**.. 12

Figure S19 ^{1}H NMR spectrum of compound **3** (400 MHz, CD_3OD)....................... 13

Figure S20 ^{13}C NMR spectrum of compound **3** (100 MHz, CD_3OD)...................... 13

Figure S21 COSY spectrum of compound **3**.. 14

Figure S22 HSQC spectrum of compound **3** 14

Figure S23 HMBC spectrum of compound **3** 15

Figure S24 NOESY spectrum of compound **3**.. 15

Figure S25 (+) HRESIMS Spectrum of compound **3**.. 16

Figure S26 IR spectrum of compound **3**.. 16

Figure S27 UV Spectrum of compound **3**.. 17

Figure S28 ^{1}H NMR spectrum of compound **4** (400 MHz, CD_3OD)....................... 17

Figure S29 ^{13}C NMR spectrum of compound **4** (100 MHz, CD_3OD)...................... 18

Figure S30 COSY spectrum of compound **4**.. 18
Figure S31 HSQC spectrum of compound **4** 19
Figure S32 HMBC spectrum of compound **4** 19
Figure S33 (+) HRESIMS Spectrum of compound **4**... 20
Figure S34 IR spectrum of compound **4**.. 20
Figure S35 UV Spectrum of compound **4**... 21
Figure S36 ^{1}H NMR spectrum of compound **5** (400 MHz, CD_3OD)........................ 22
Figure S37 ^{13}C NMR spectrum of compound **5** (100 MHz, CD_3OD)....................... 22
Figure S38 ^{1}H NMR spectrum of compound **6** (400 MHz, CD_3OD)........................ 23
Figure S39 ^{13}C NMR spectrum of compound **6** (100 MHz, CD_3OD)....................... 23
Figure S40 ^{1}H NMR spectrum of compound **7** (400 MHz, CD_3OD)........................ 24
Figure S41 ^{13}C NMR spectrum of compound **7** (100 MHz, CD_3OD)....................... 24
Figure S42 ^{1}H NMR spectrum of compound **8** (400 MHz, CD_3OD)........................ 25
Figure S43 ^{13}C NMR spectrum of compound **8** (100 MHz, CD_3OD)....................... 25
Figure S44 ^{1}H NMR spectrum of compound **9** (400 MHz, CD_3OD)........................ 26
Figure S45 ^{13}C NMR spectrum of compound **9** (100 MHz, CD_3OD)....................... 26
S1. Computational Section 27

Figure S1 ^{1}H NMR spectrum of compound **1** (600 MHz, CD_3OD)

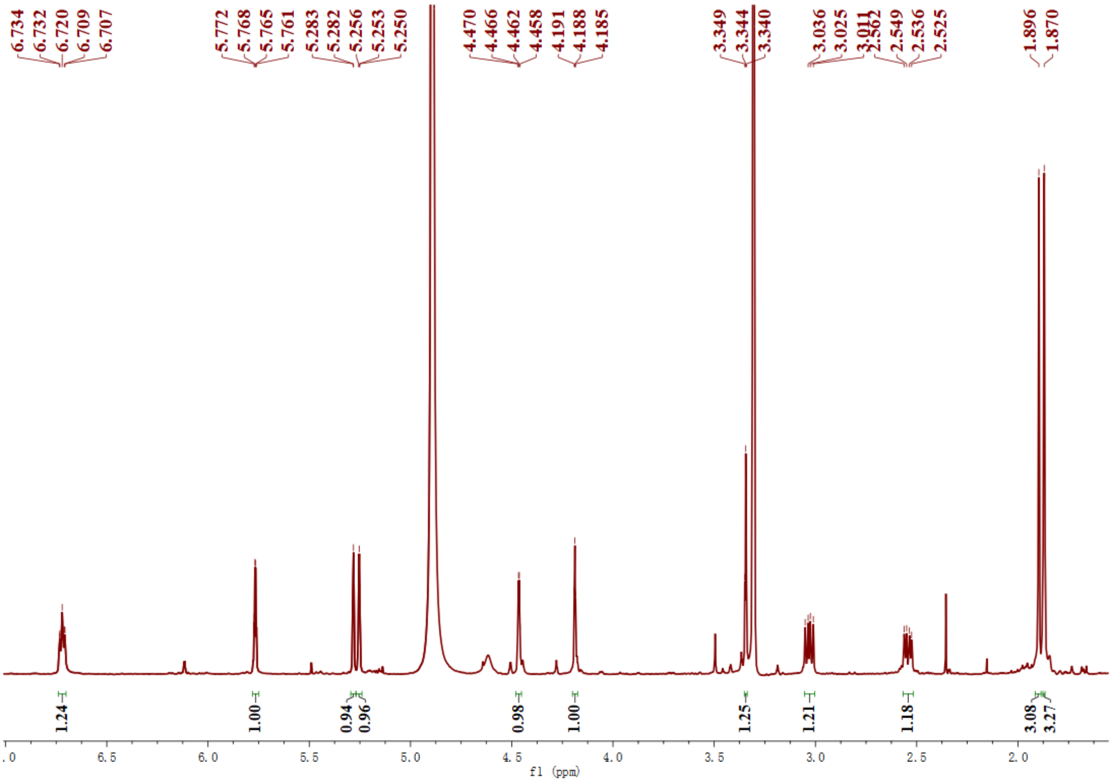

Figure S2 ^{13}C NMR spectrum of compound **1** (150 MHz, CD_3OD)

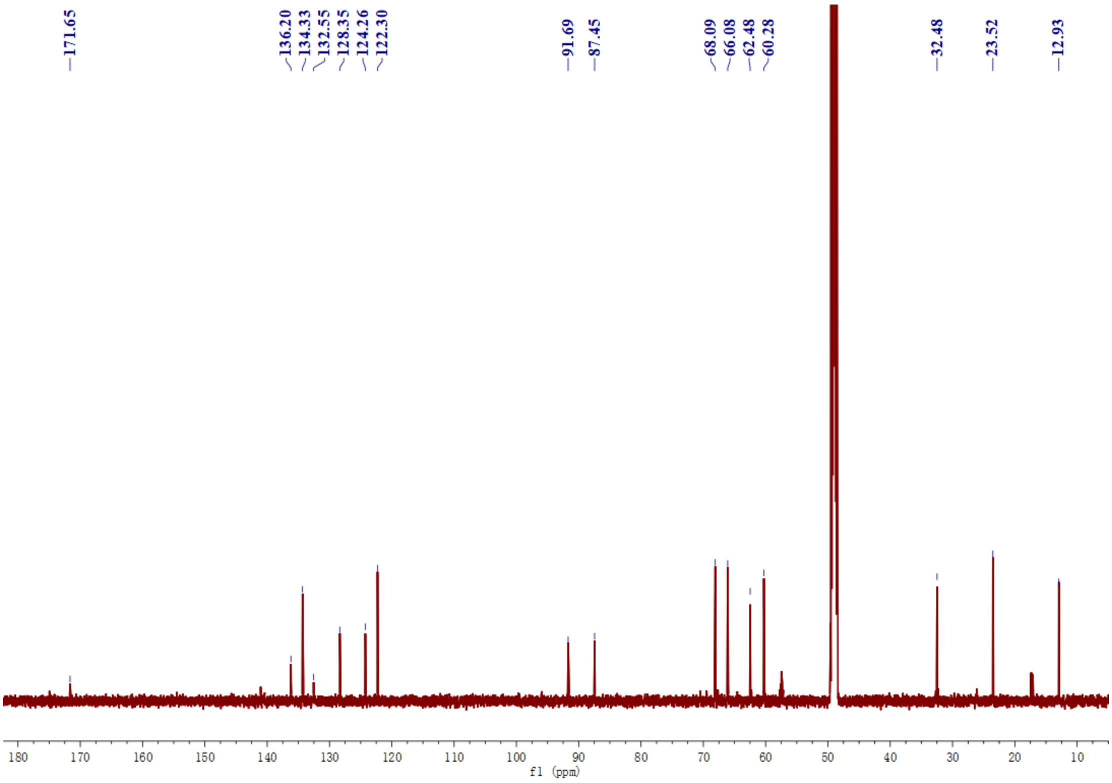

Figure S3 COSY spectrum of compound **1**

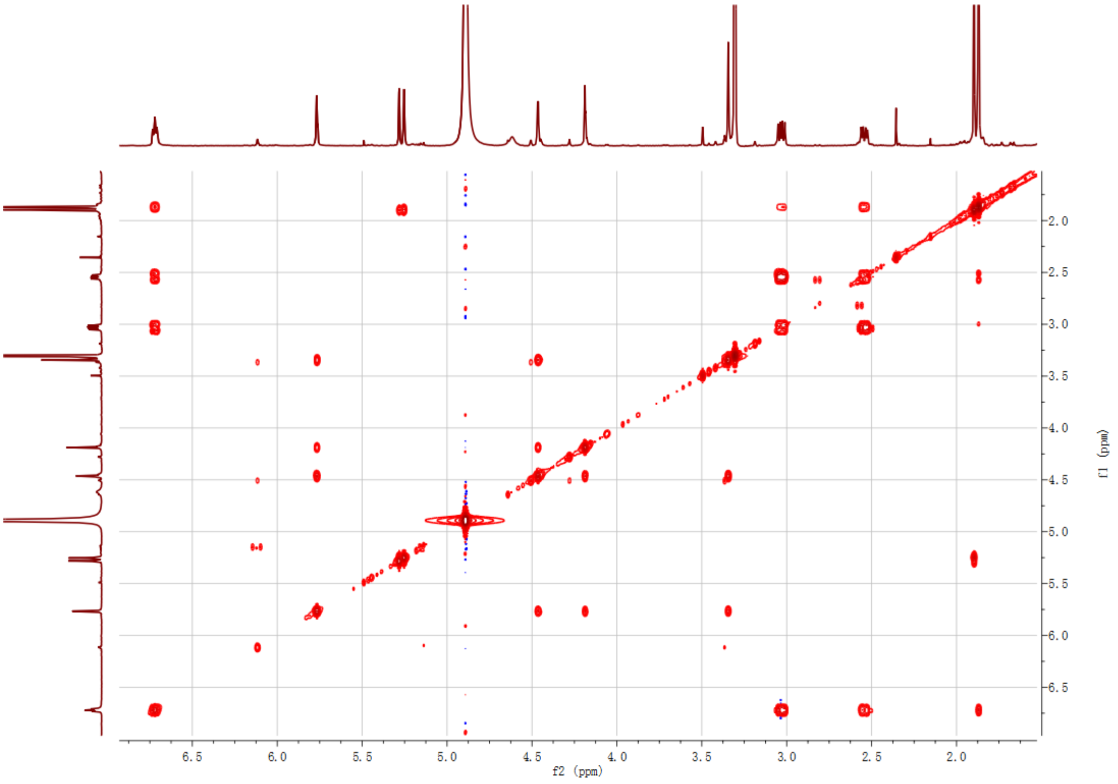

Figure S4 HSQC spectrum of compound **1**

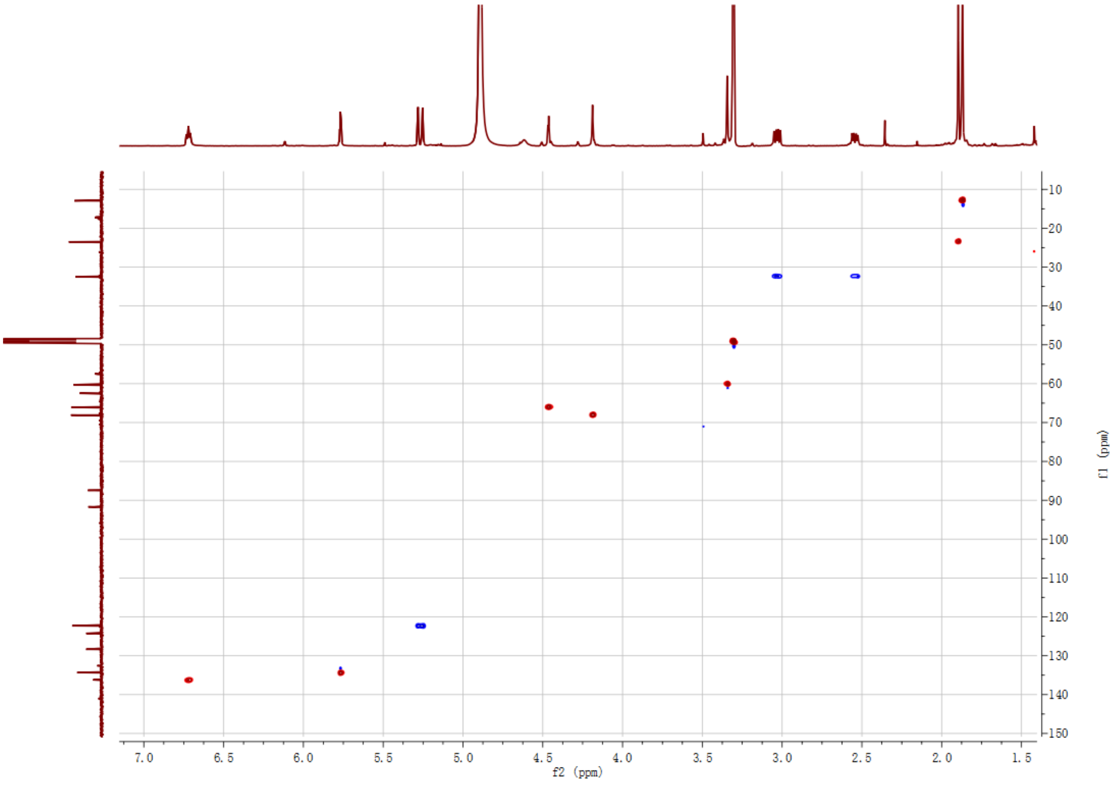

Figure S5 HMBC spectrum of compound **1**

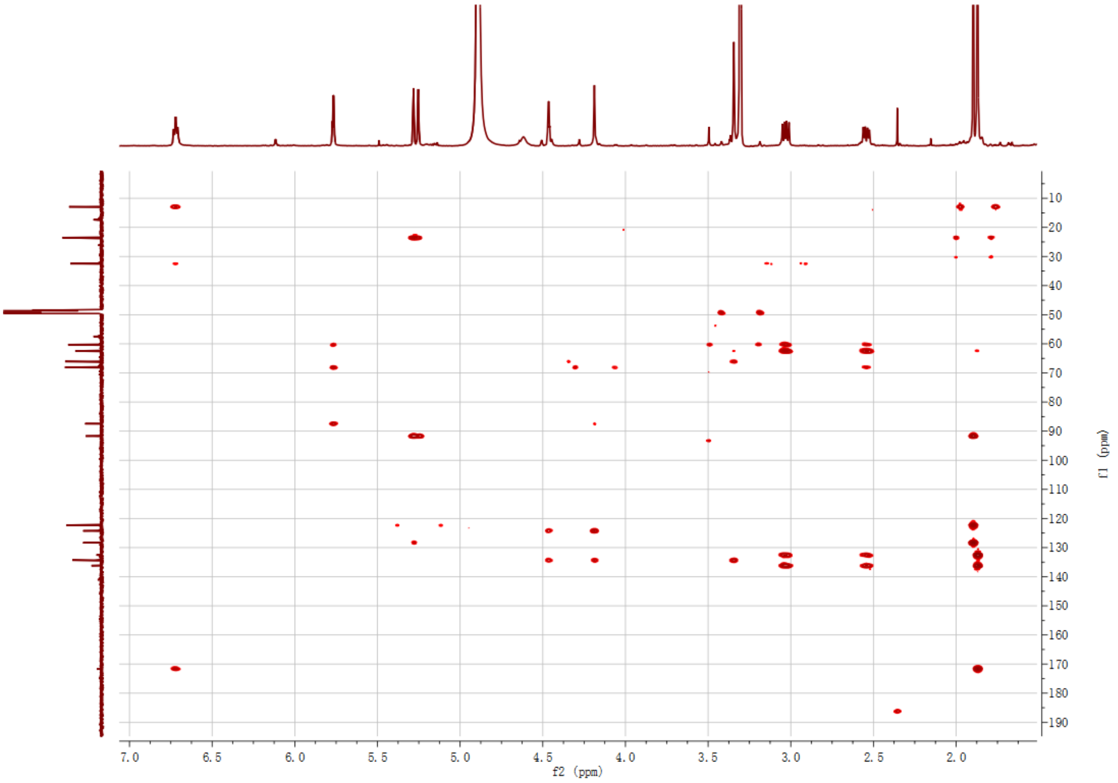

Figure S6 NOESY spectrum of compound **1**

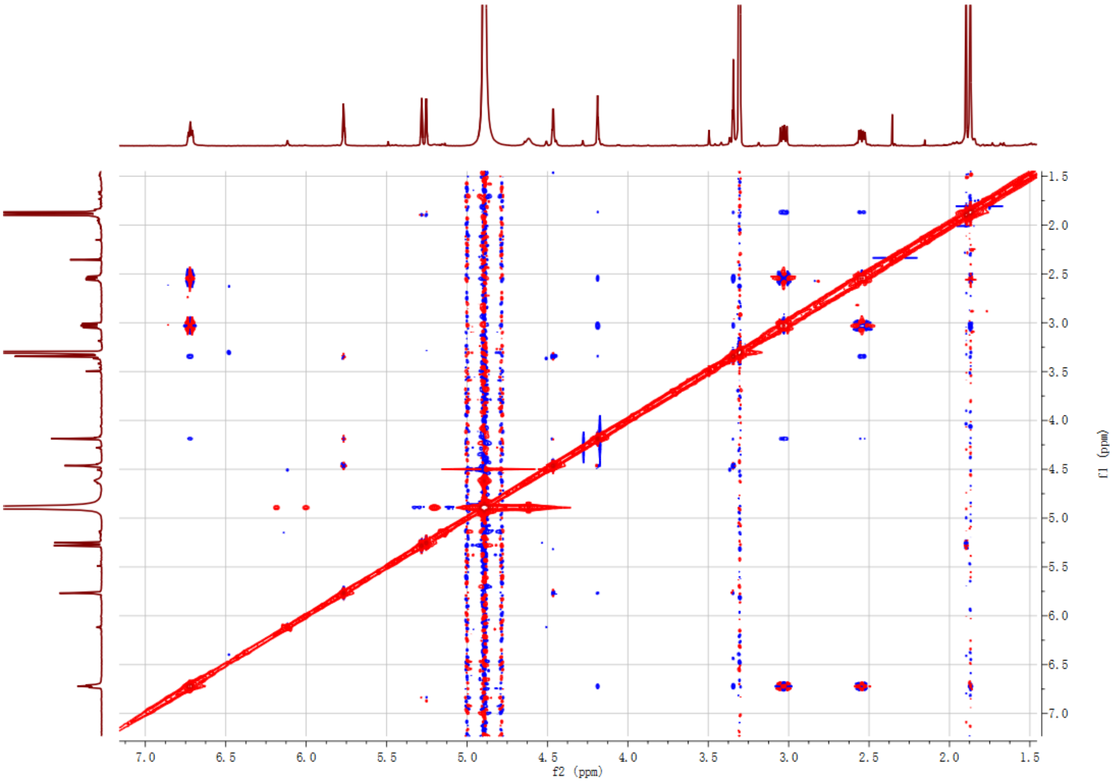

Figure S7 (+) HRESIMS Spectrum of compound **1**

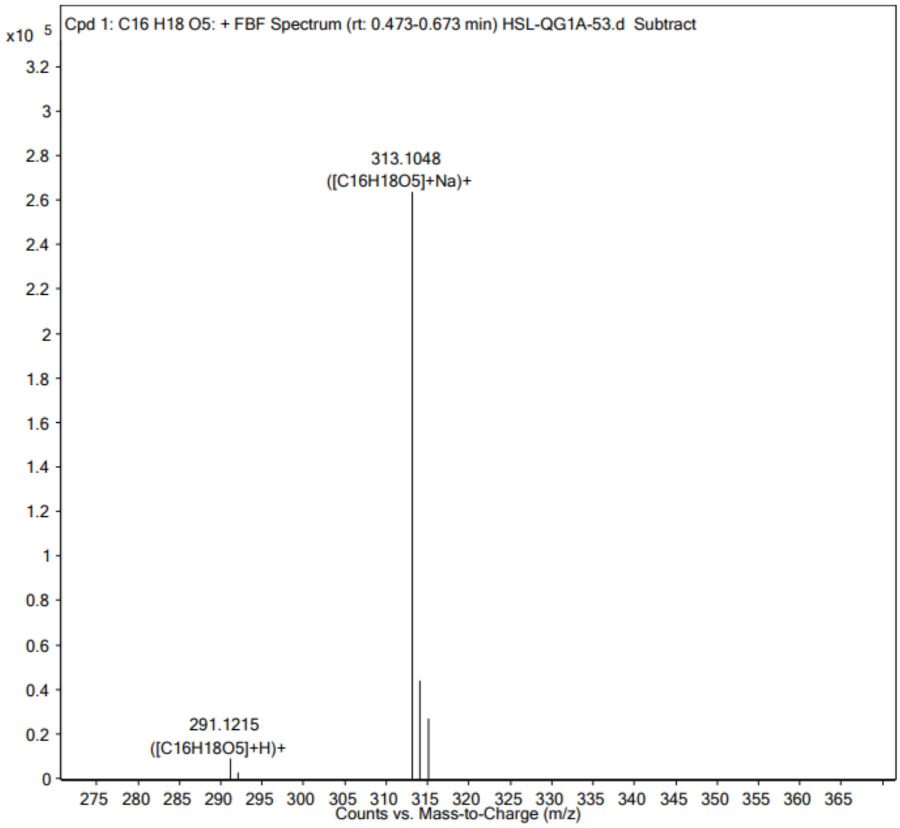

Figure S8 IR spectrum of compound **1**

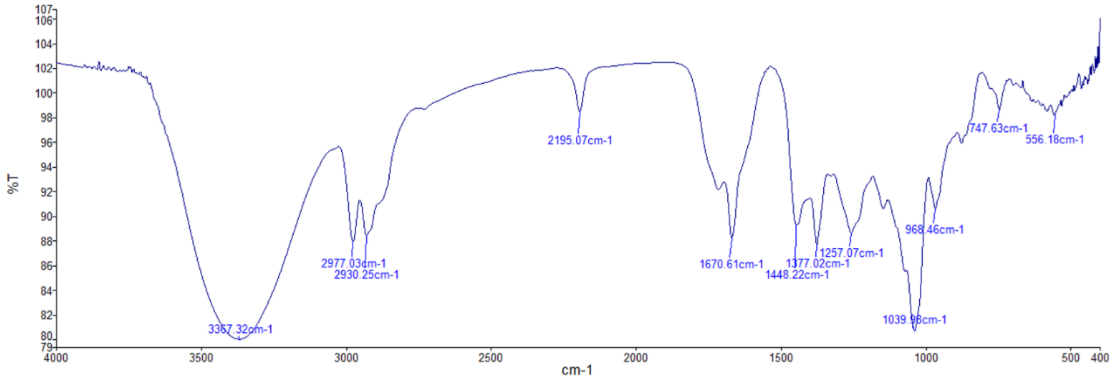

Figure S9 UV Spectrum of compound **1**

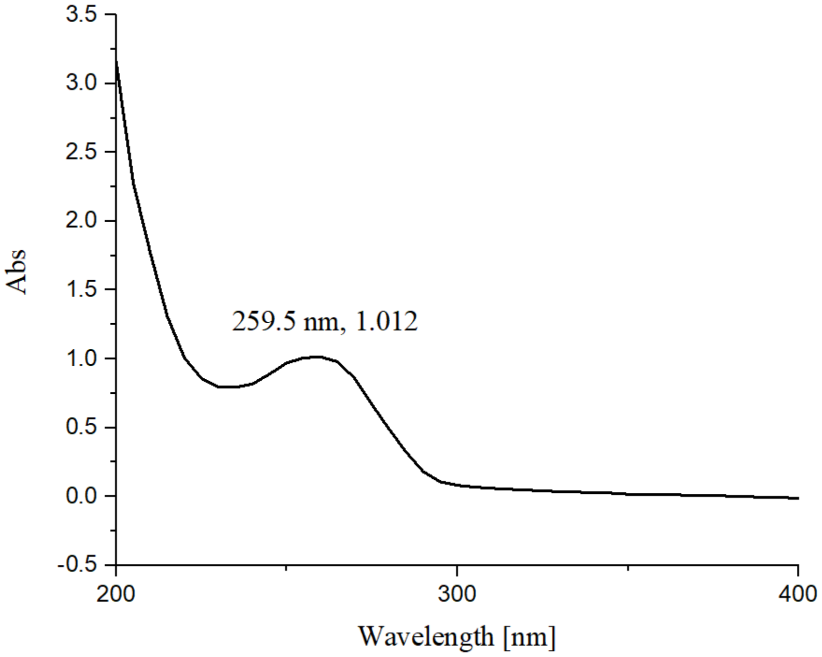

Figure S10 ^{1}H NMR spectrum of compound **2** (400 MHz, CD_3OD)

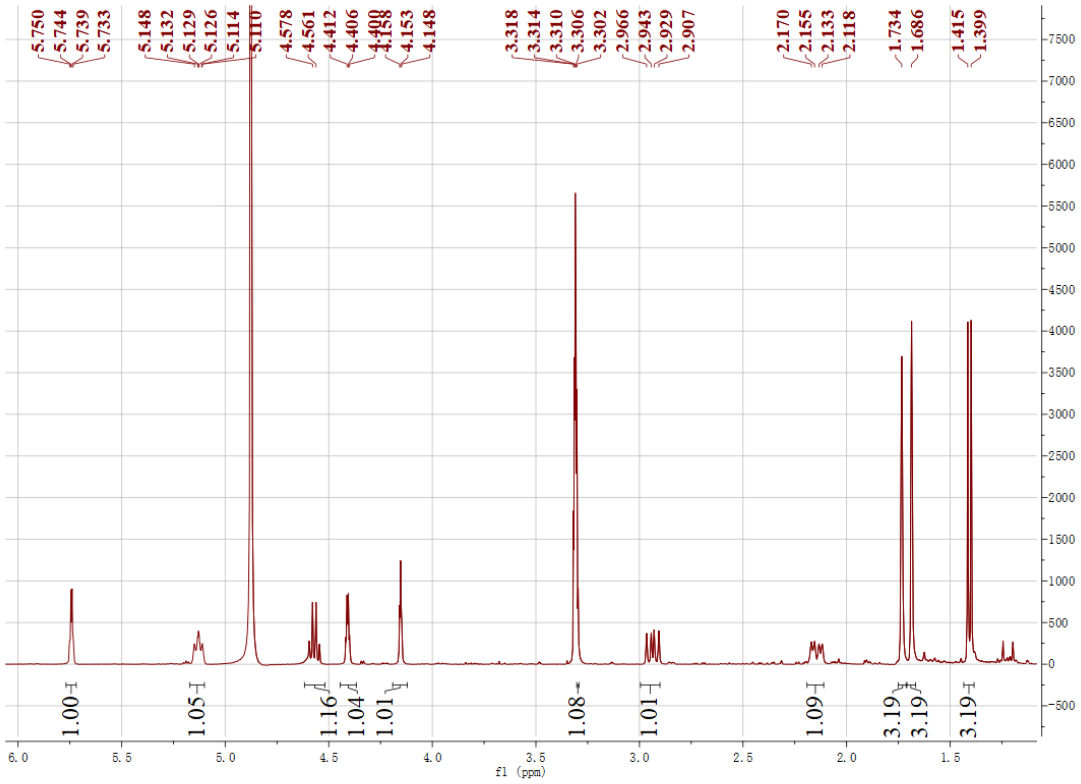

Figure S11 ^{13}C NMR spectrum of compound **2** (100 MHz, CD_3OD)

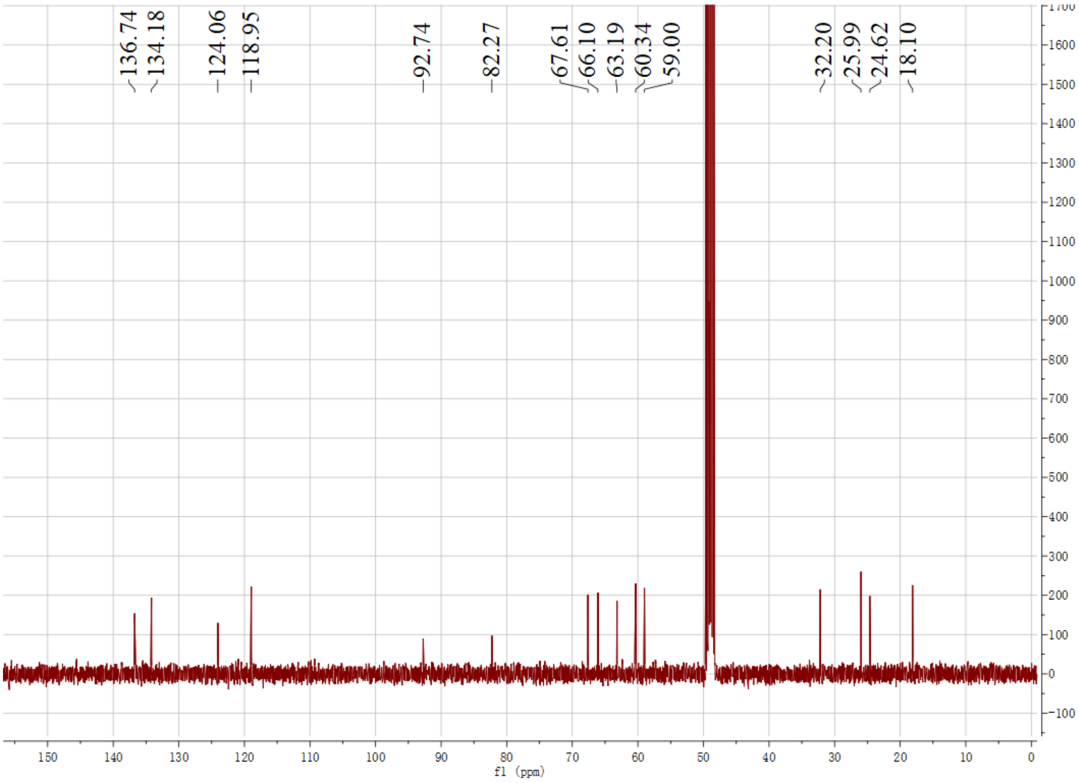

Figure S12 COSY spectrum of compound **2**

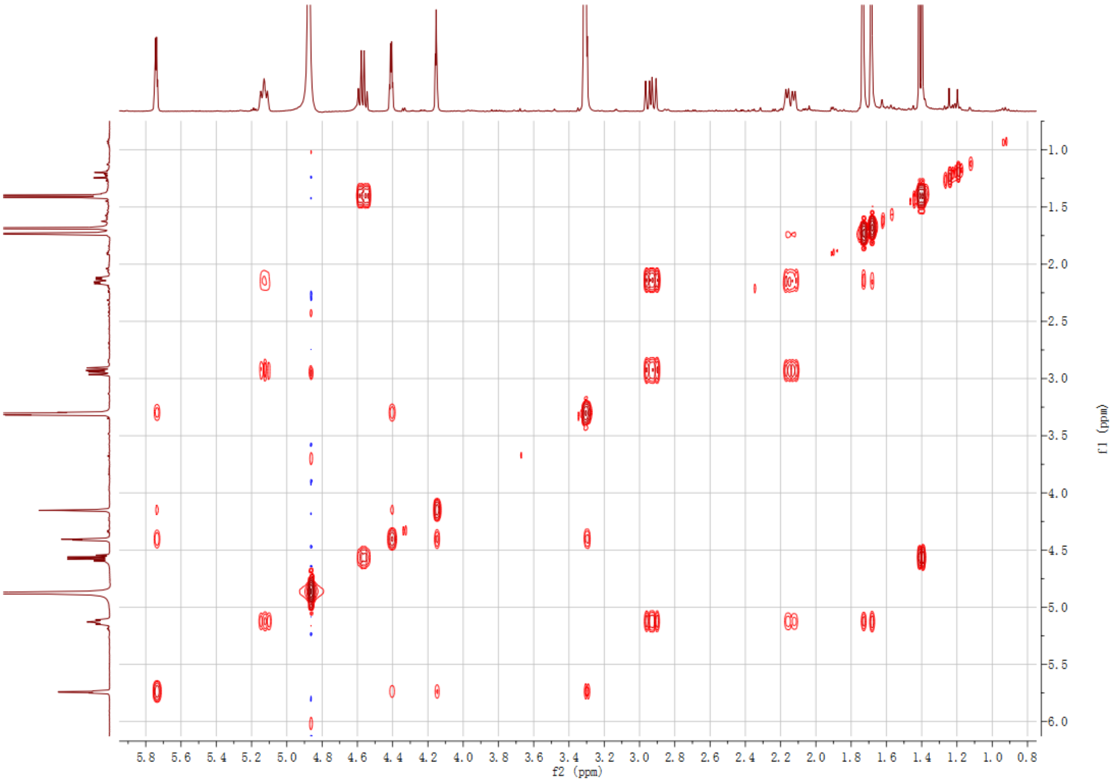

Figure S13 HSQC spectrum of compound **2**

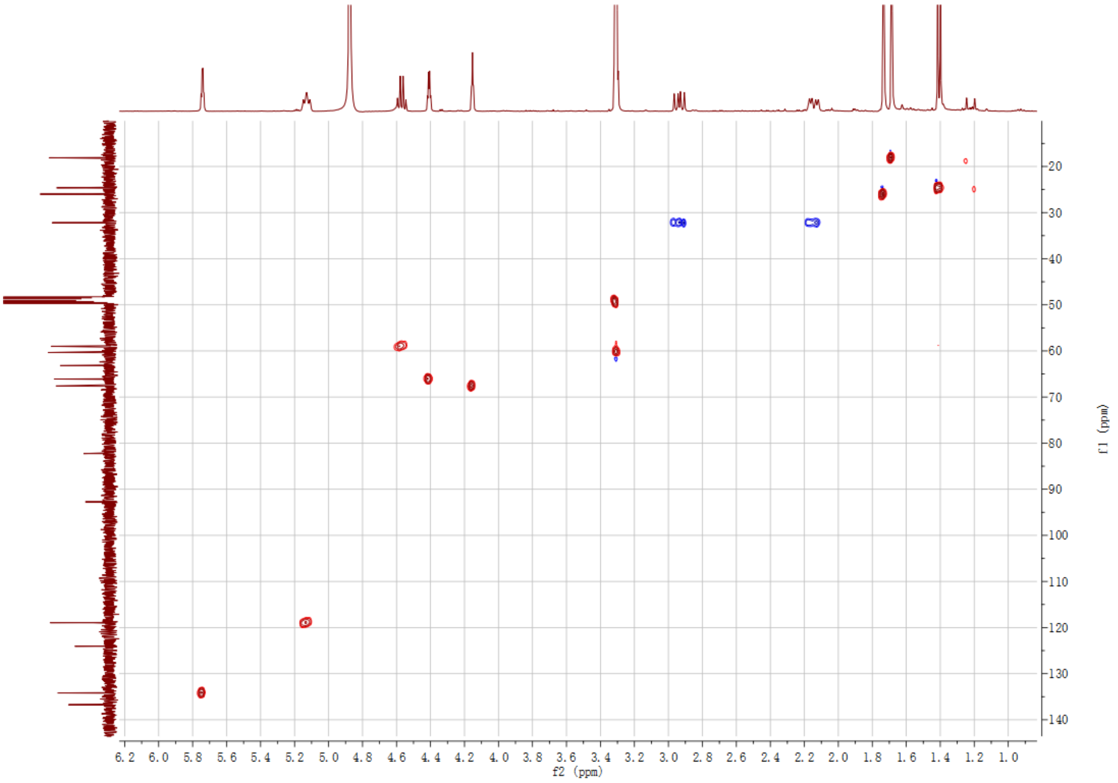

Figure S14 HMBC spectrum of compound **2**

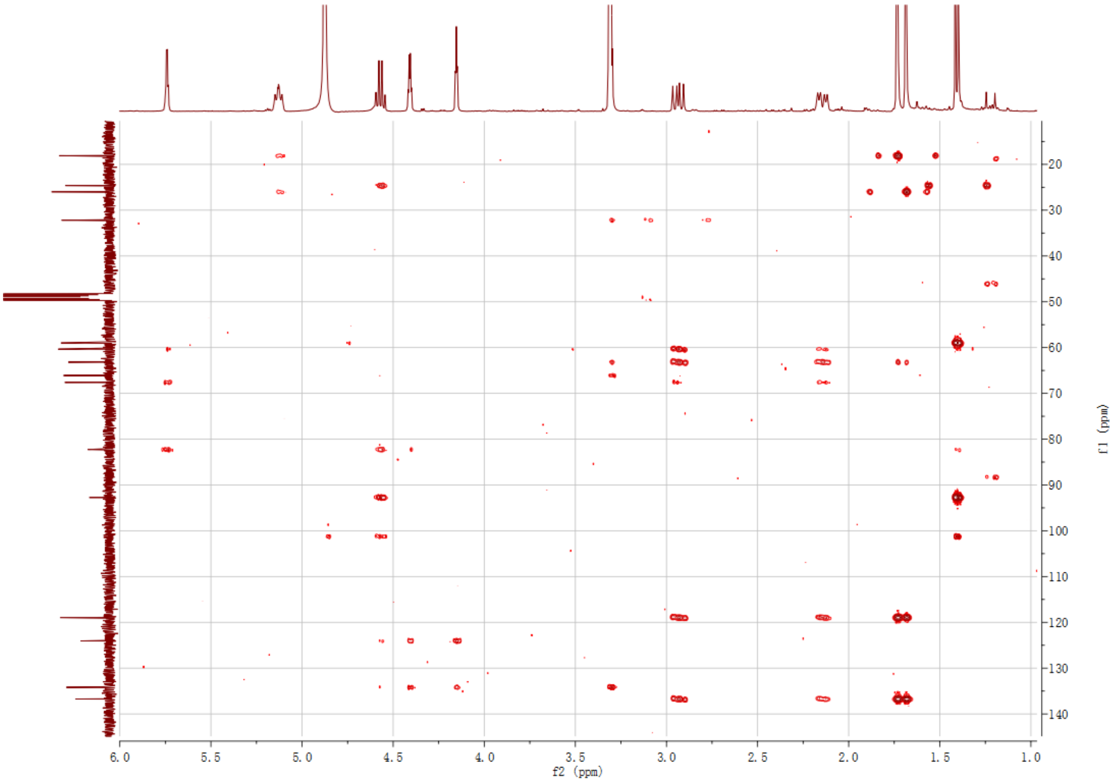

Figure S15 NOESY spectrum of compound **2**

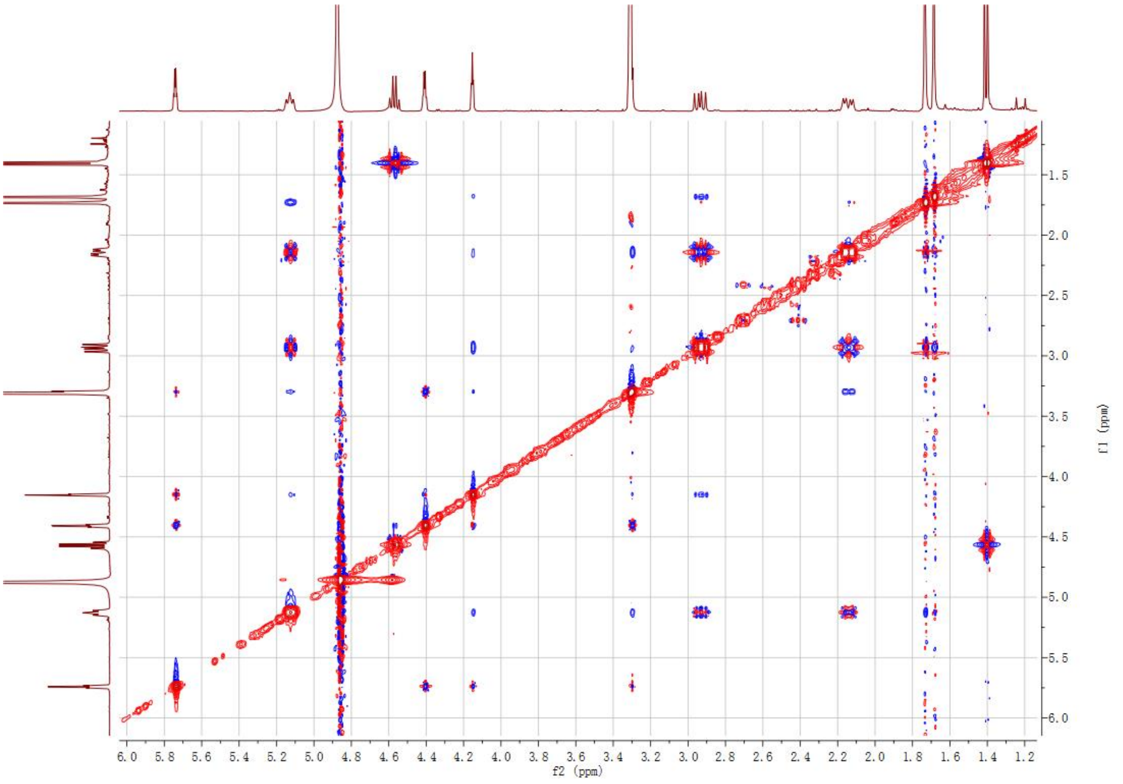

Figure S16 (+) HRESIMS Spectrum of compound **2**

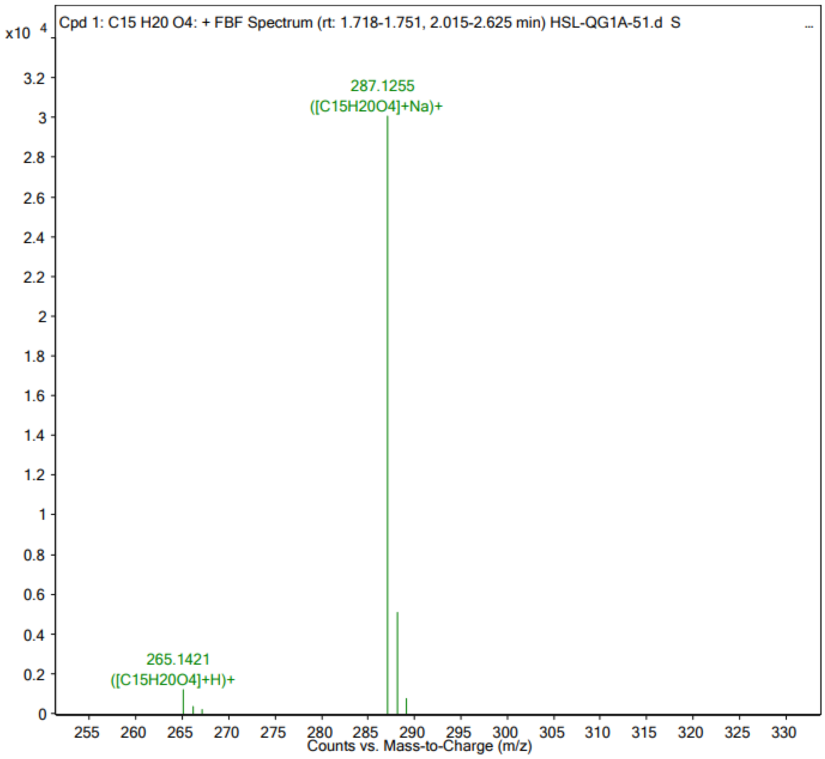

Figure S17 IR spectrum of compound **2**

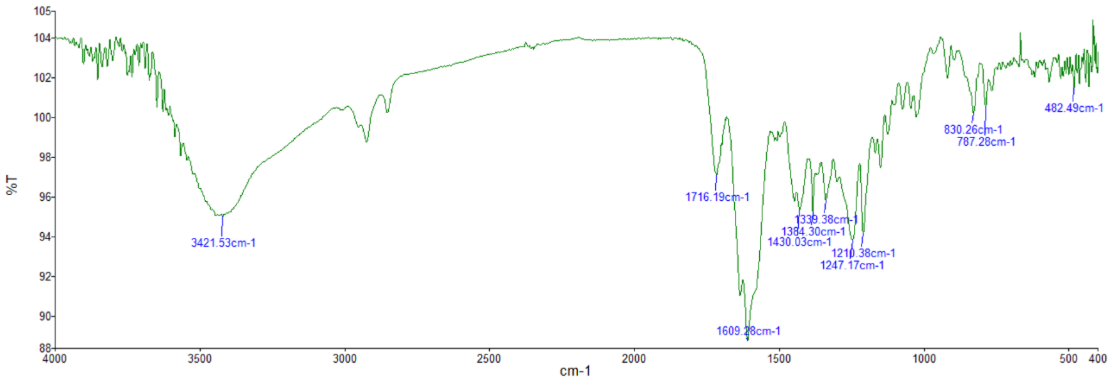

Figure S18 UV Spectrum of compound **2**

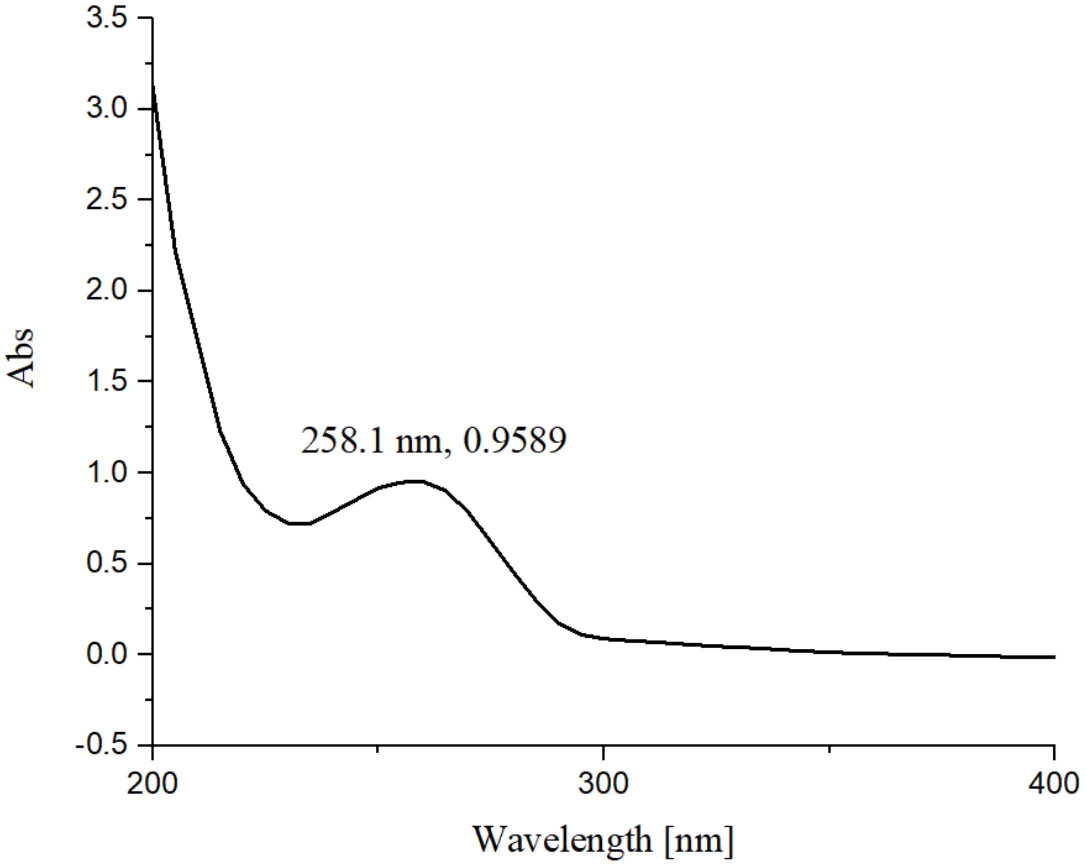

Figure S19 ^{1}H NMR spectrum of compound **3** (400 MHz, CD_3OD)

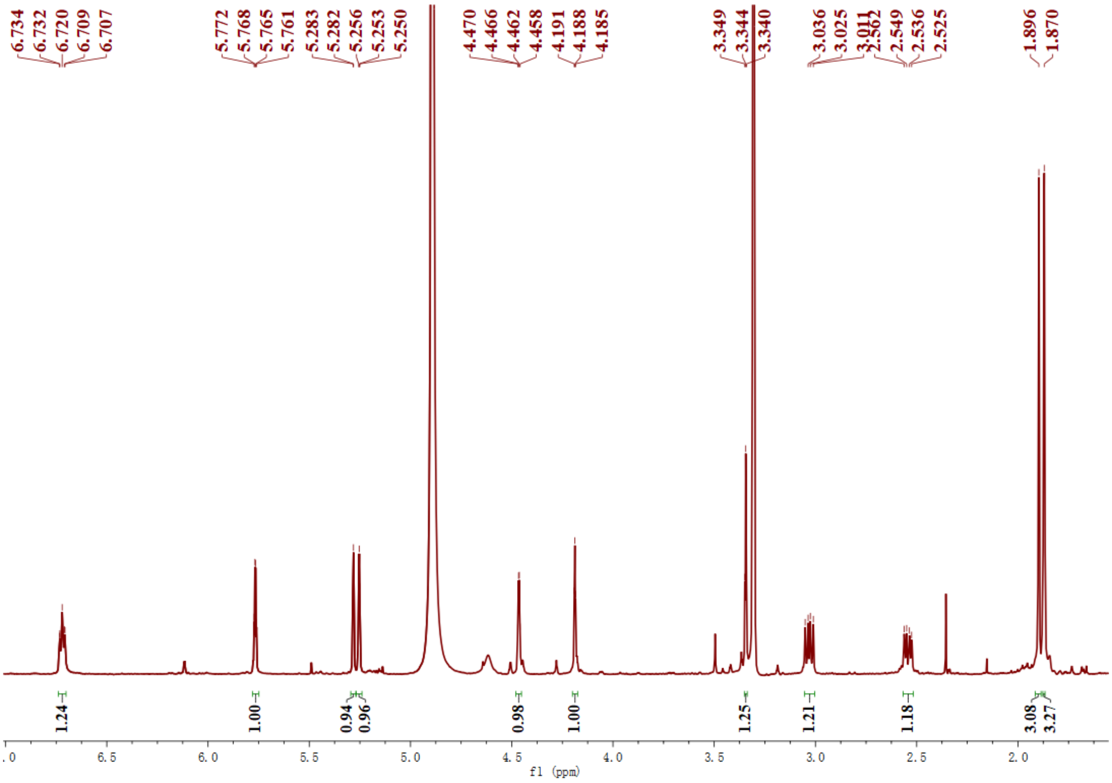

Figure S20 ^{13}C NMR spectrum of compound **3** (100 MHz, CD_3OD)

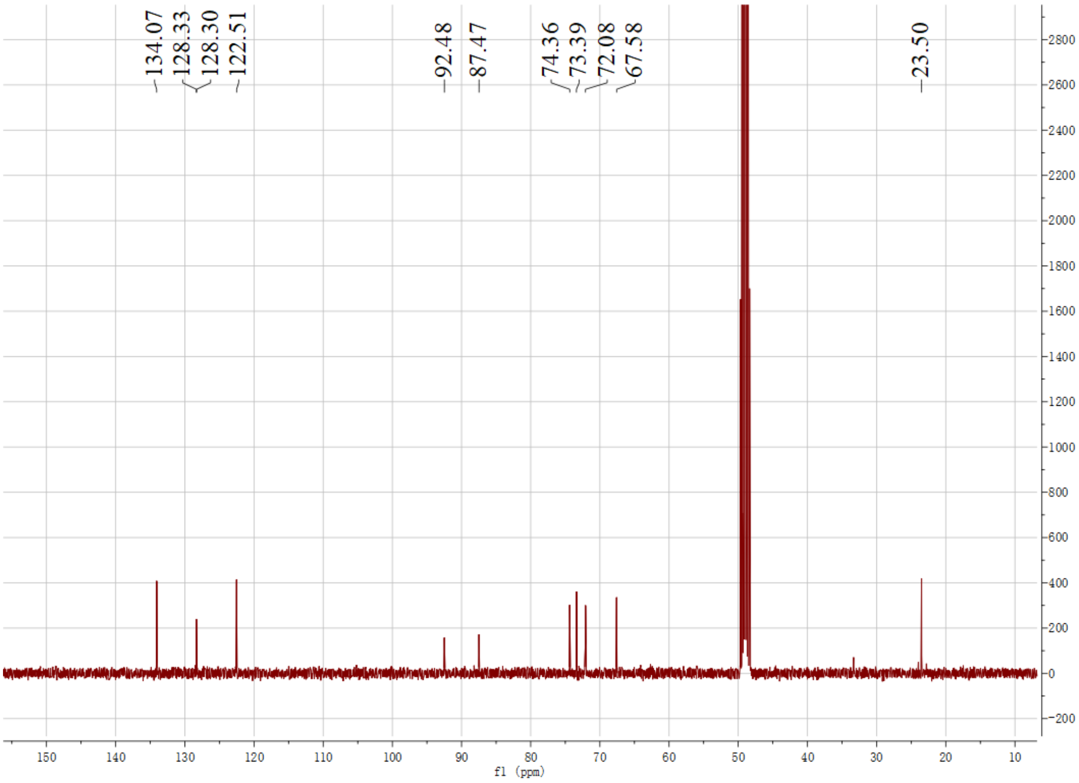

Figure S21 COSY spectrum of compound **3**

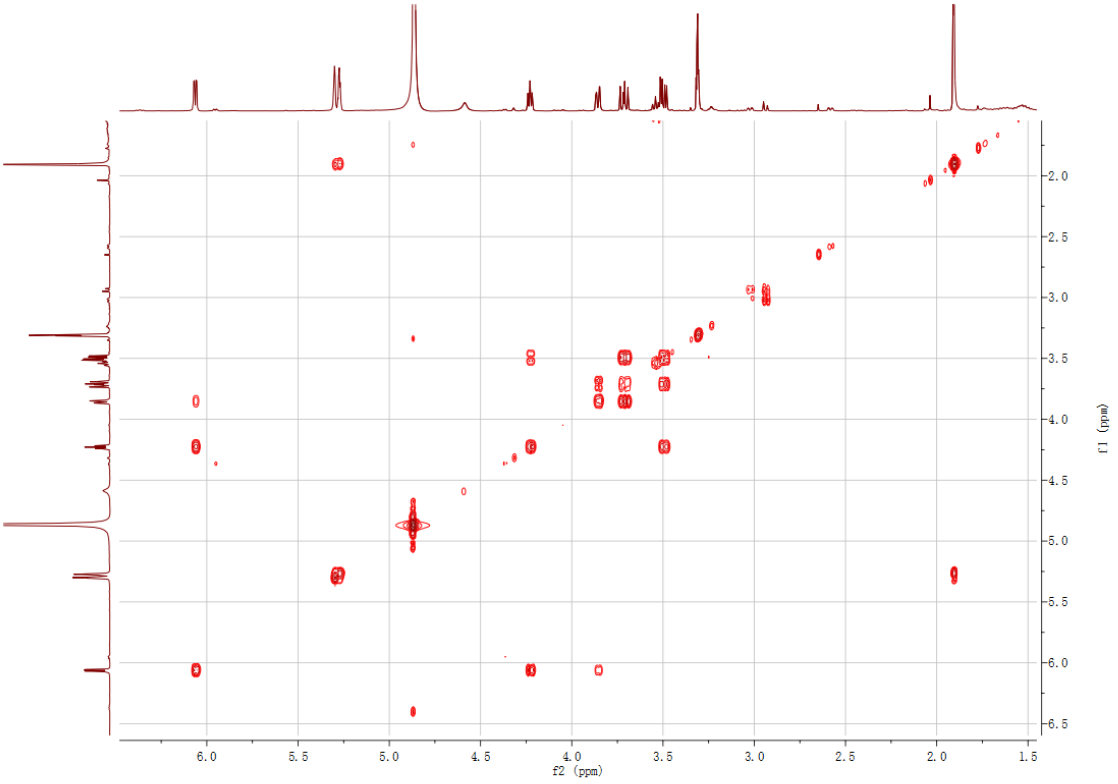

Figure S22 HSQC spectrum of compound **3**

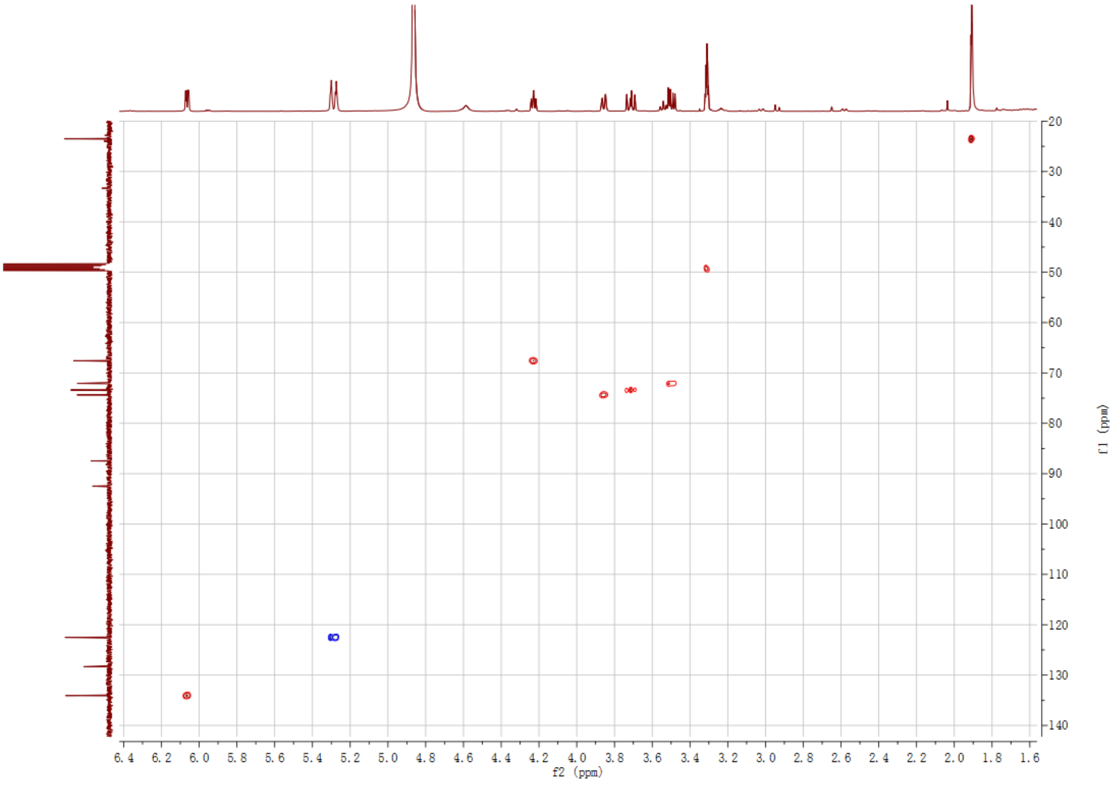

Figure S23 HMBC spectrum of compound **3**

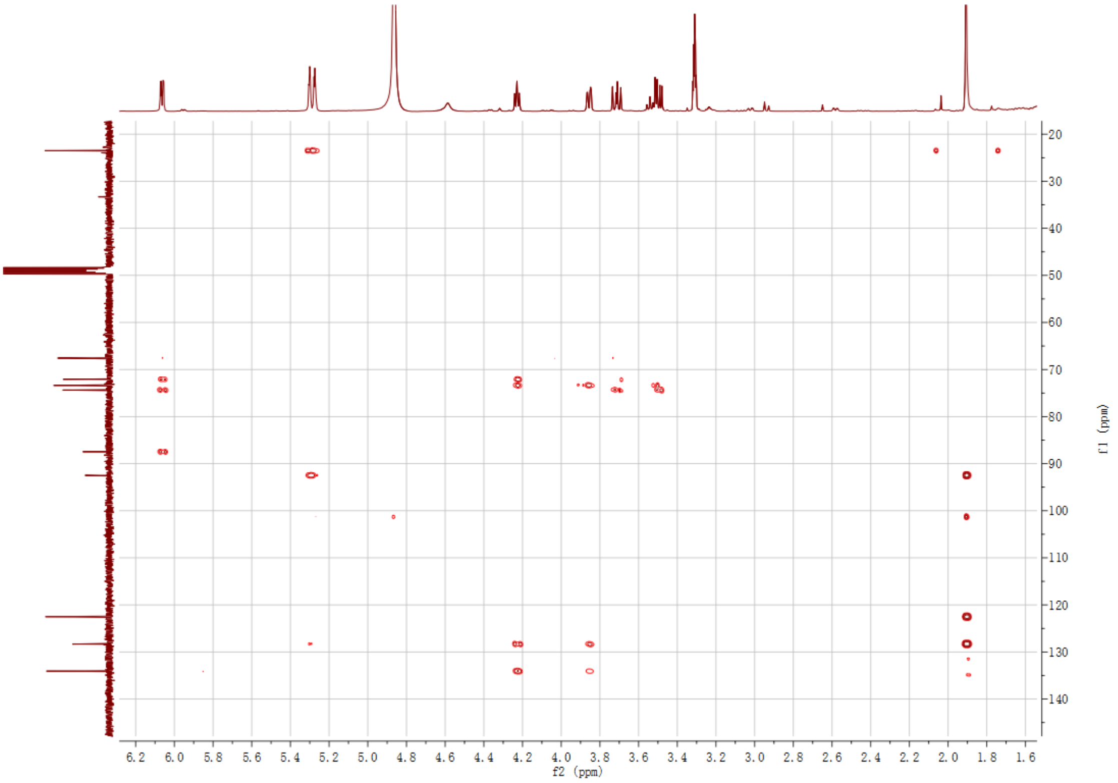

Figure S24 NOESY spectrum of compound **3**

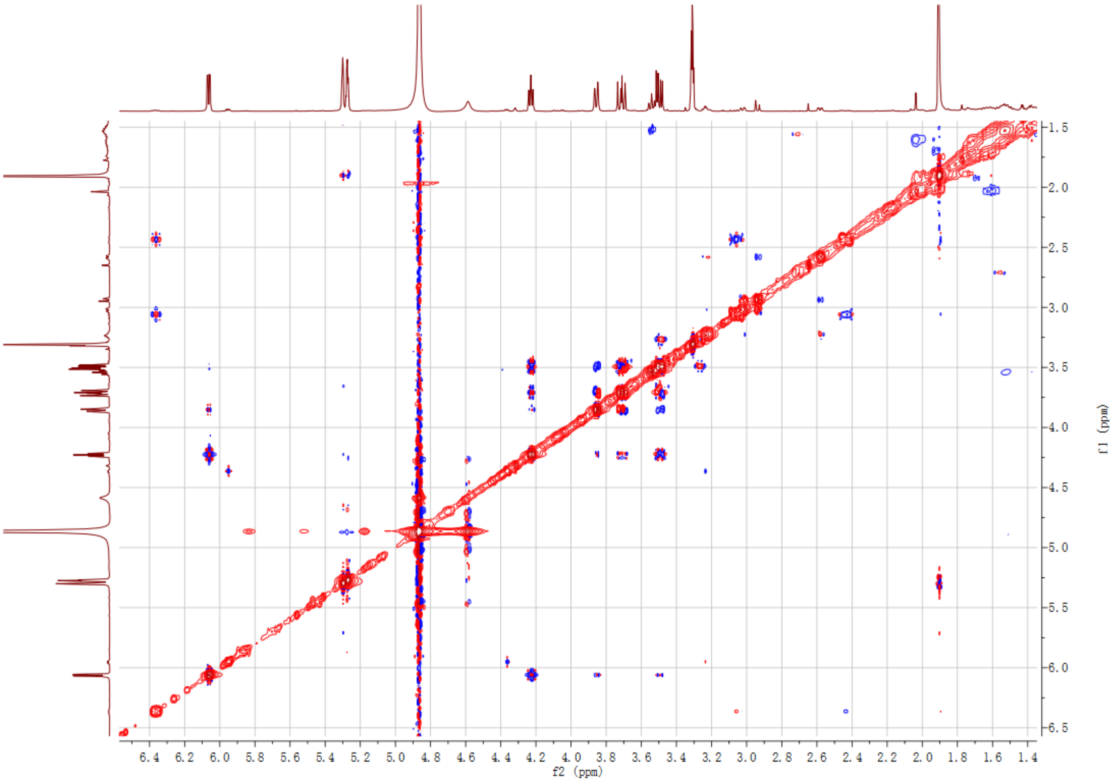

Figure S25 (+) HRESIMS Spectrum of compound **3**

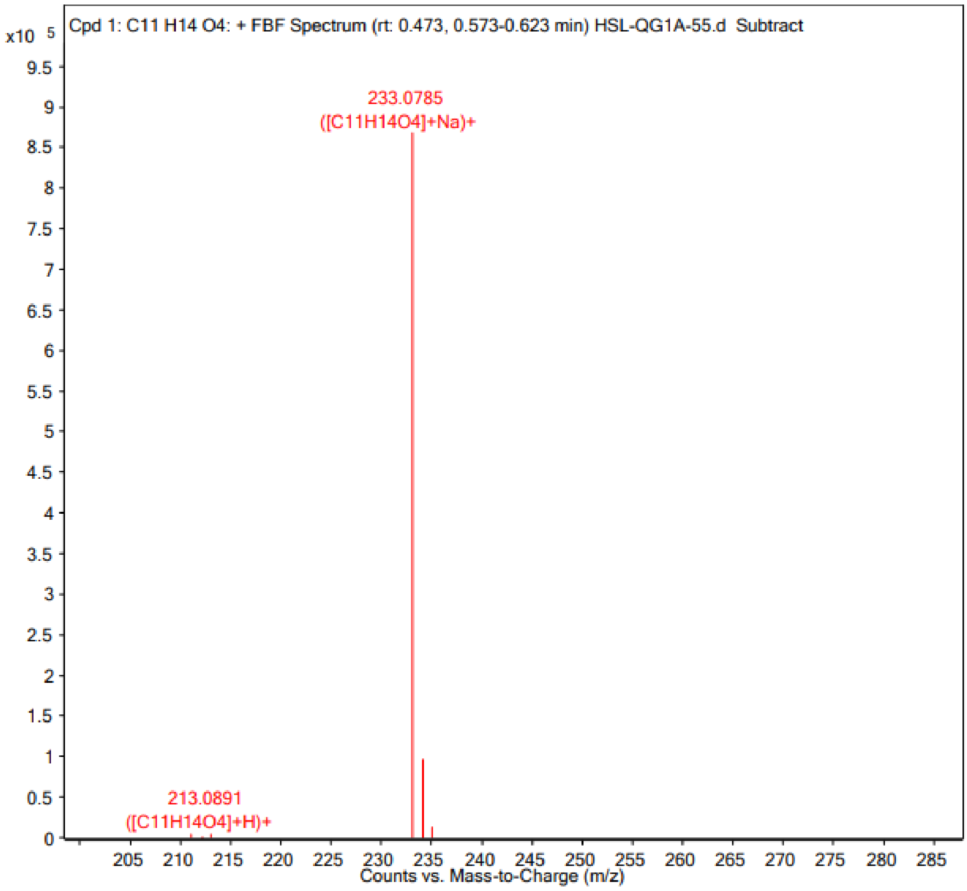

Figure S26 IR spectrum of compound **3**

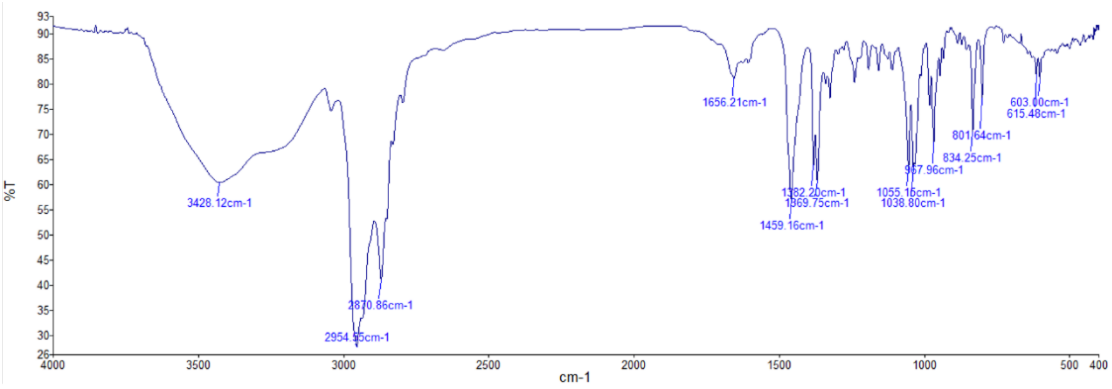

Figure S27 UV Spectrum of compound **3**

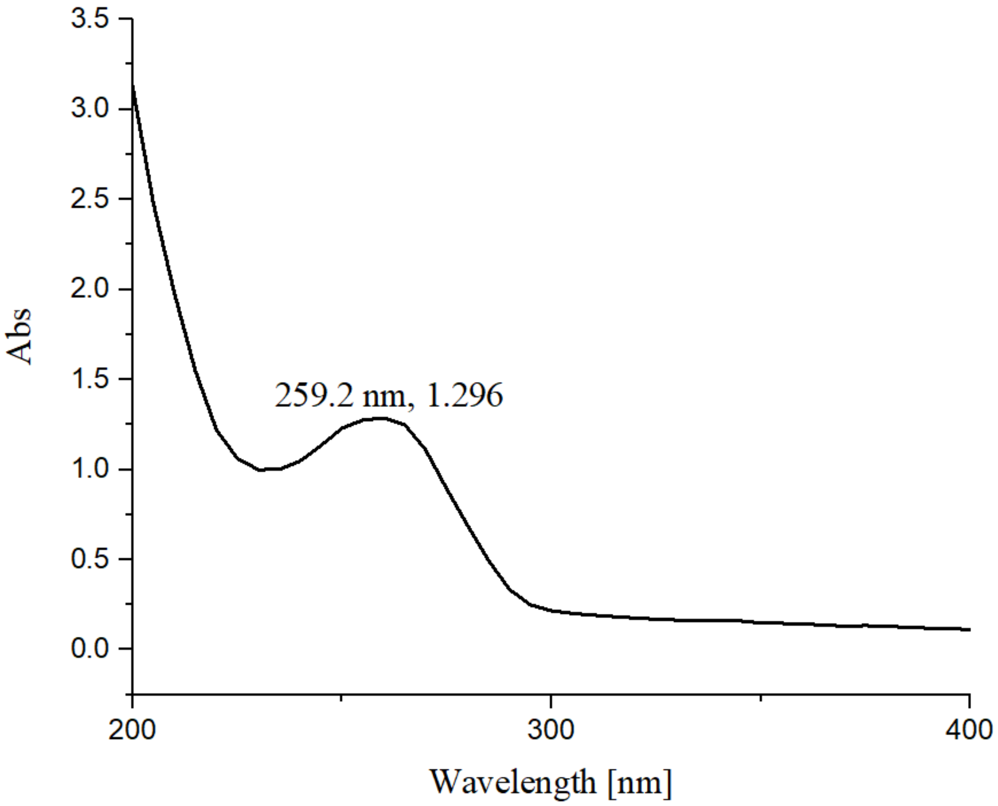

Figure S28 ^{1}H NMR spectrum of compound **4** (400 MHz, CD_3OD)

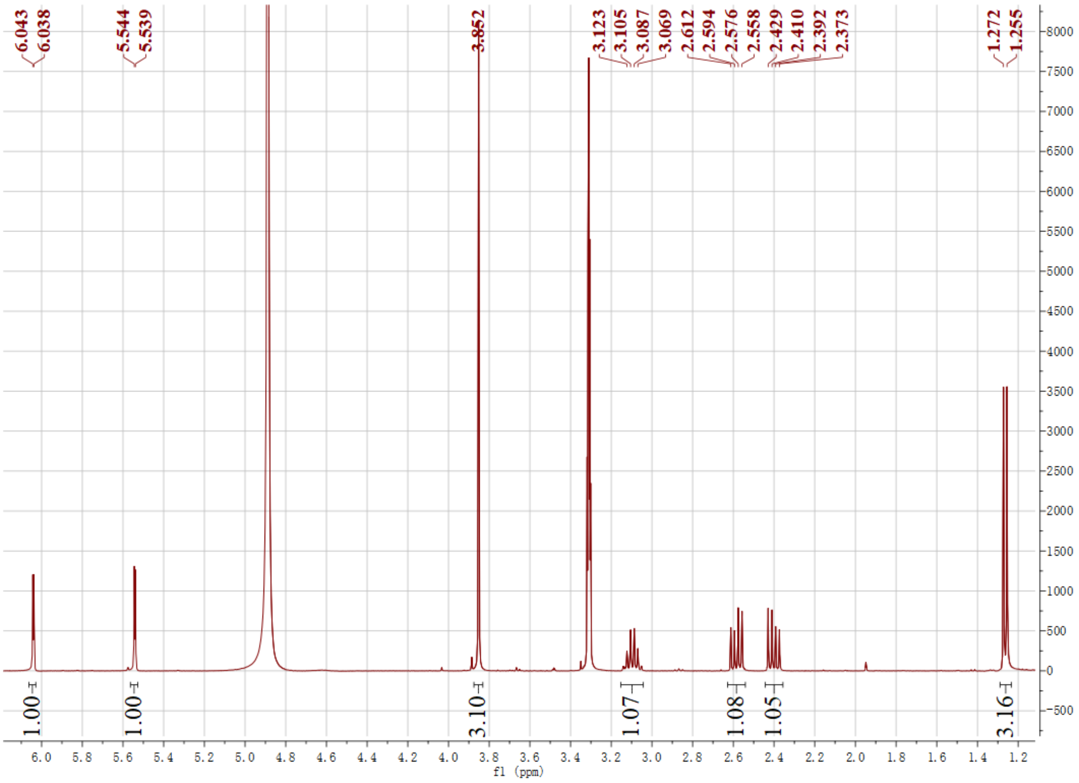

Figure S29 ^{13}C NMR spectrum of compound **4** (100 MHz, CD_3OD)

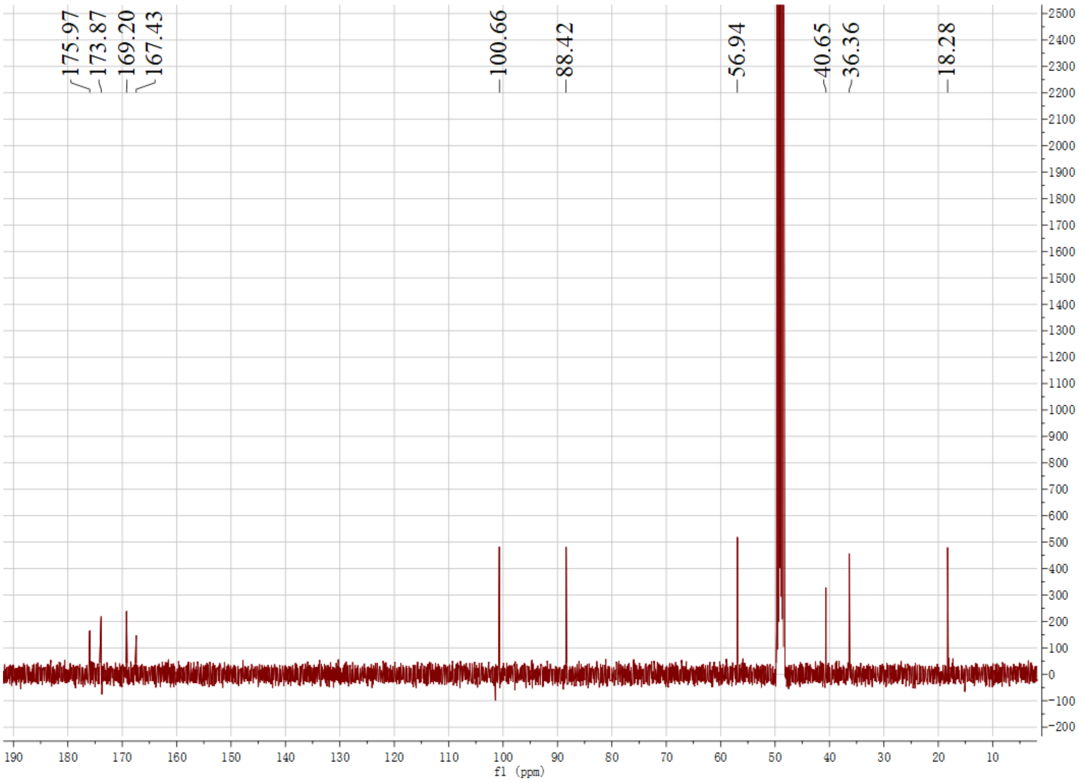

Figure S30 COSY spectrum of compound **4**

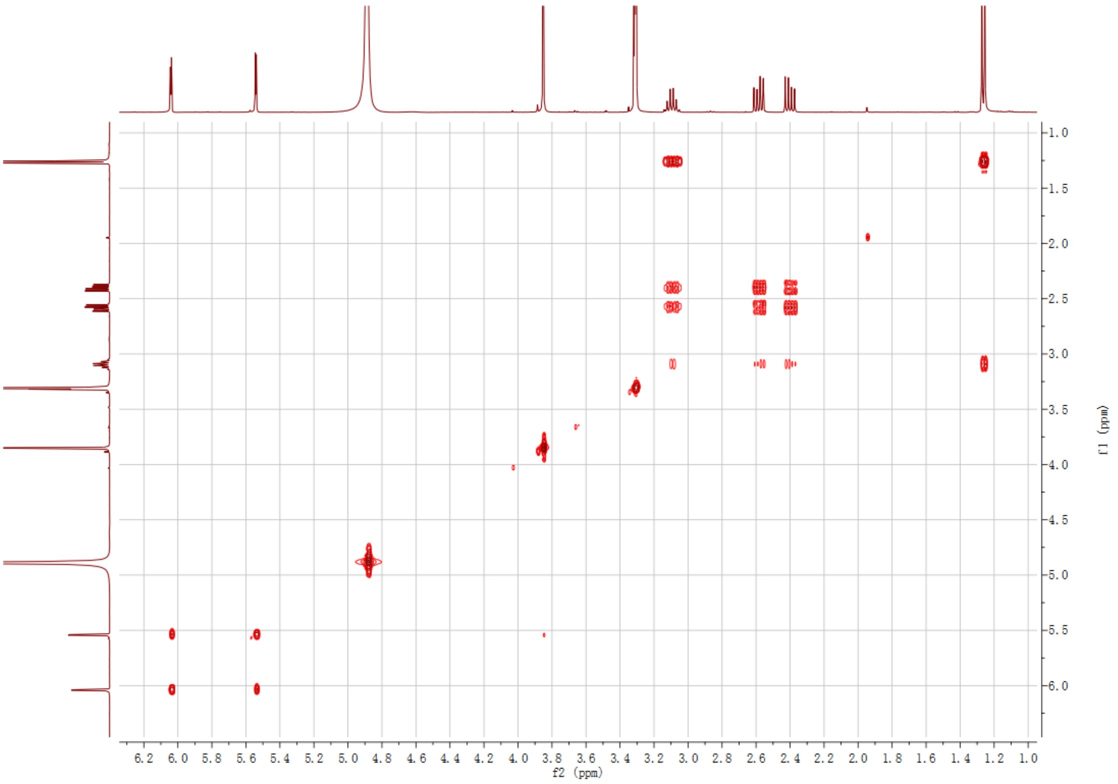

Figure S31 HSQC spectrum of compound **4**

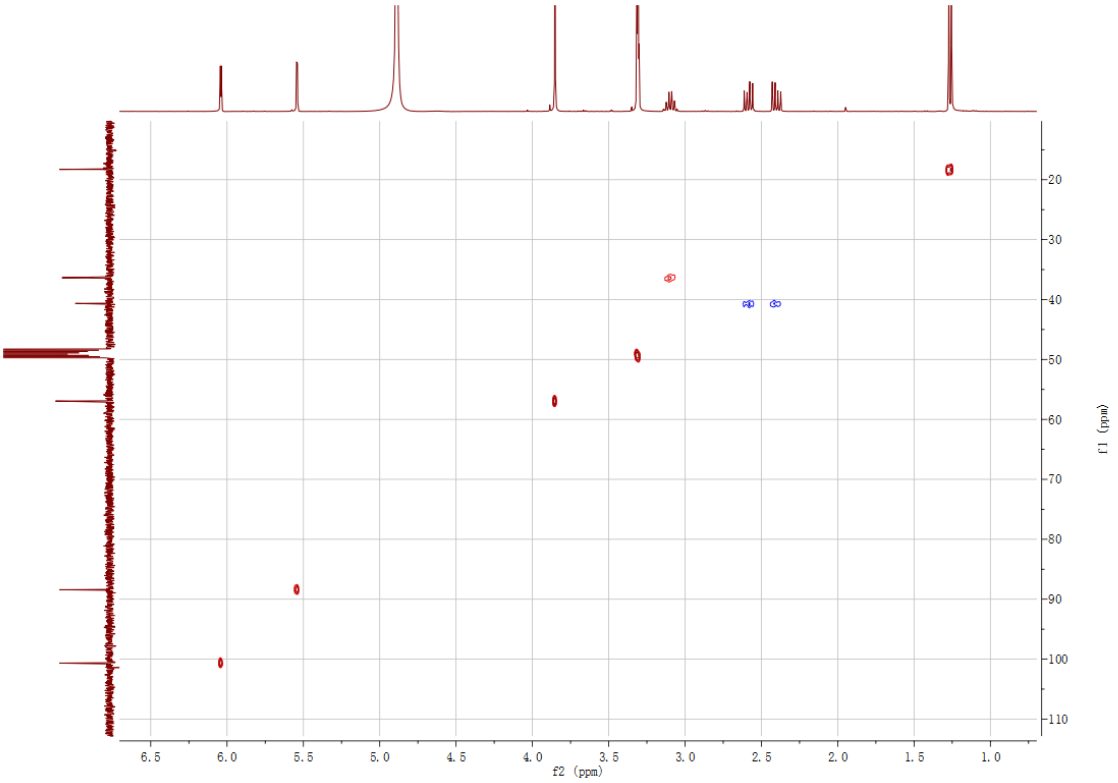

Figure S32 HMBC spectrum of compound **4**

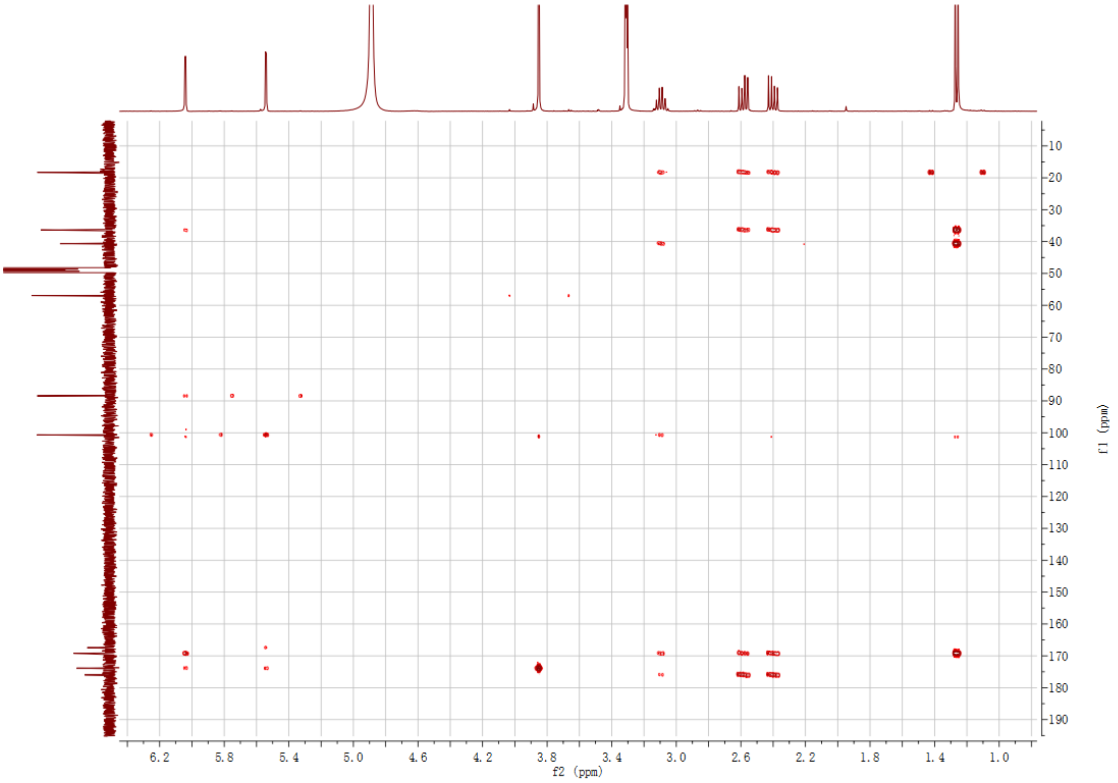

Figure S33 (+) HRESIMS Spectrum of compound **4**

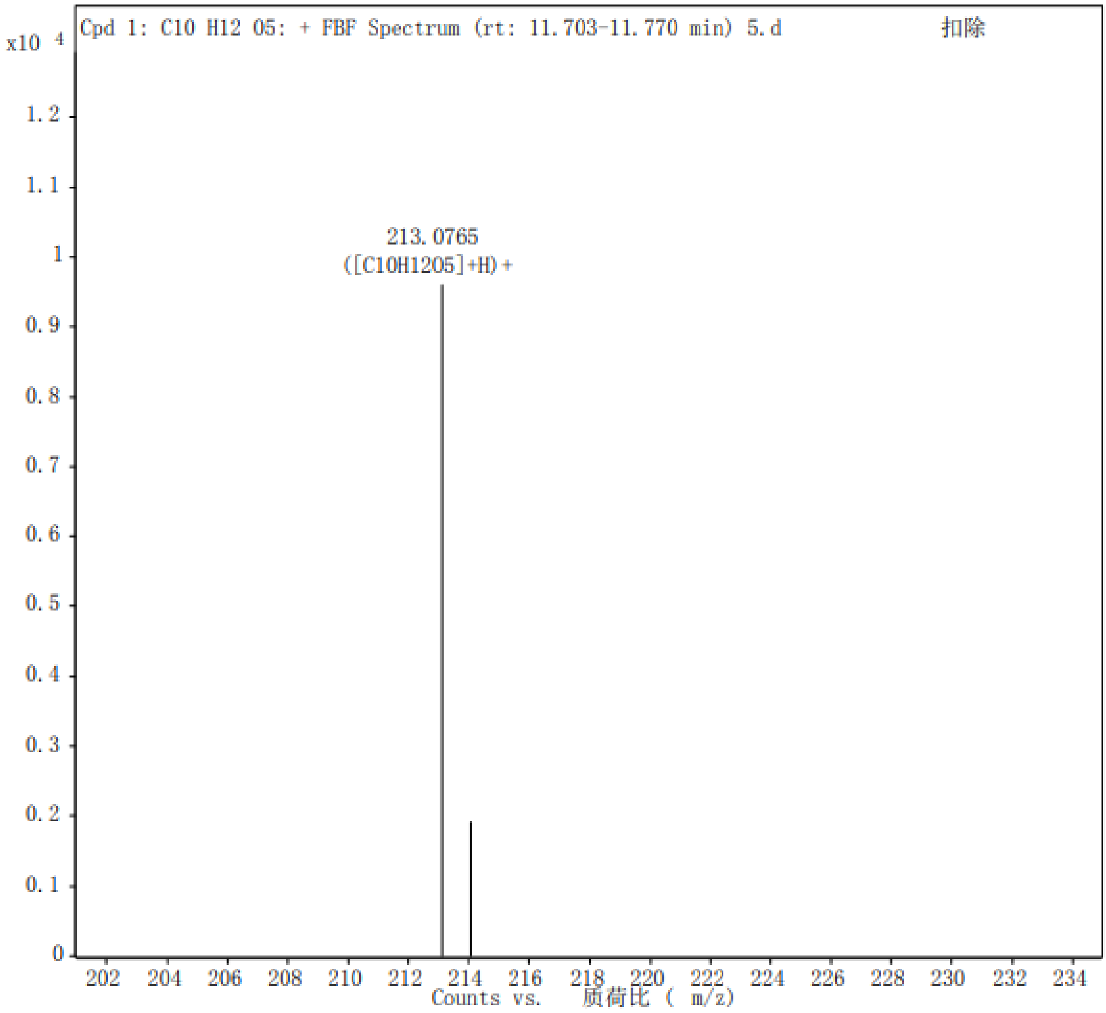

Figure S34 IR spectrum of compound **4**

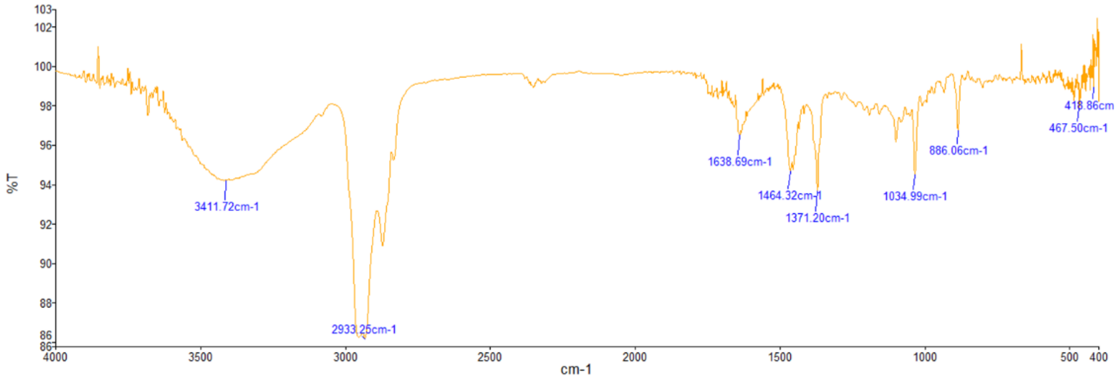

Figure S35 UV Spectrum of compound **4**

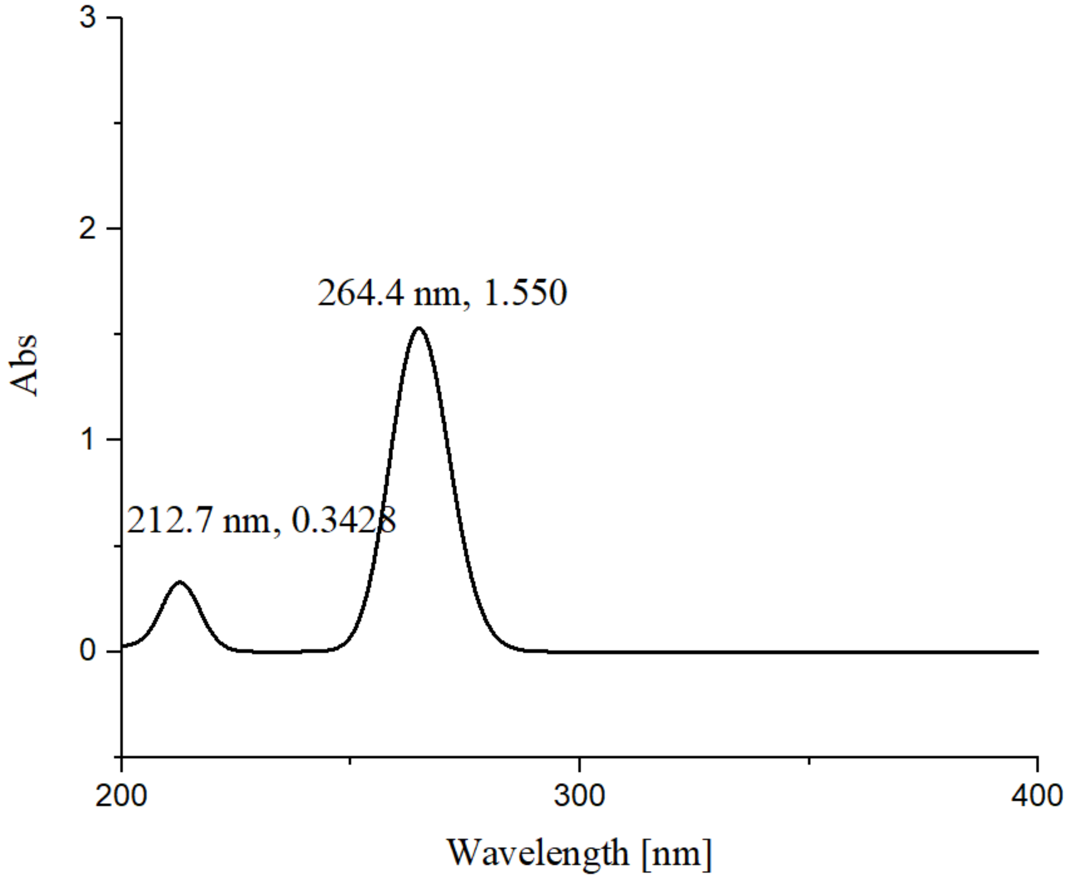

Figure S36 ^{1}H NMR spectrum of compound **5** (400 MHz, CD_3OD)

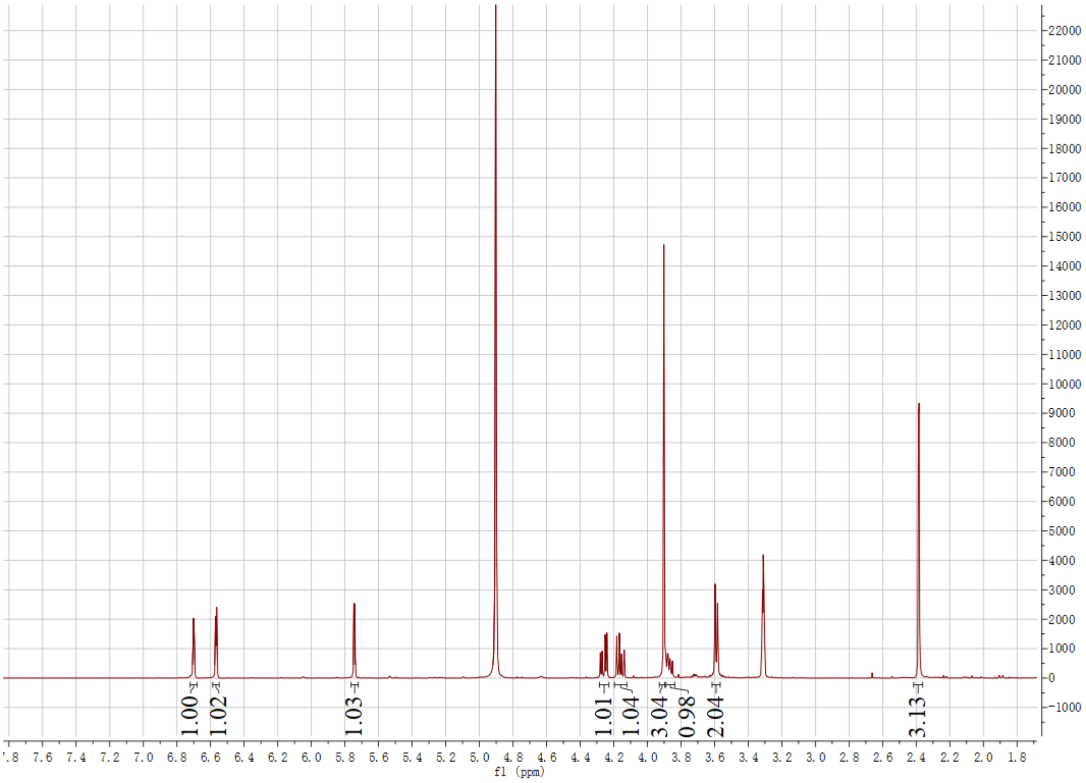

Figure S37 ^{13}C NMR spectrum of compound **5** (100 MHz, CD_3OD)

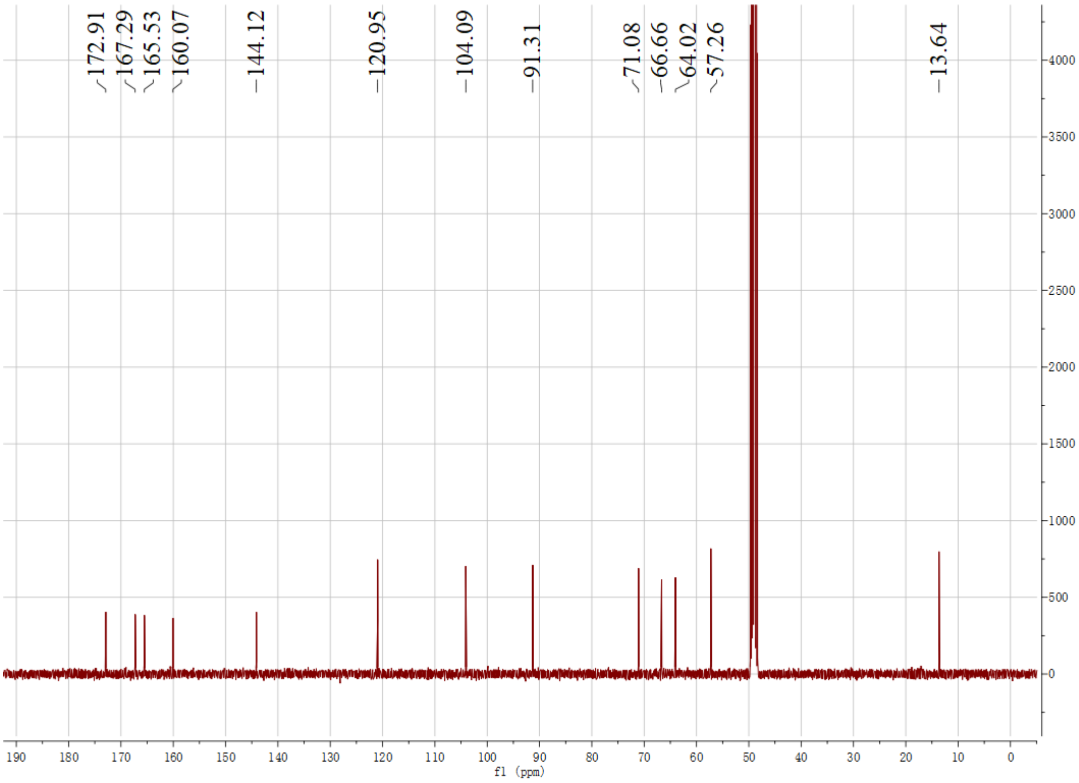

Figure S38 ^{1}H NMR spectrum of compound **6** (400 MHz, CD_3OD)

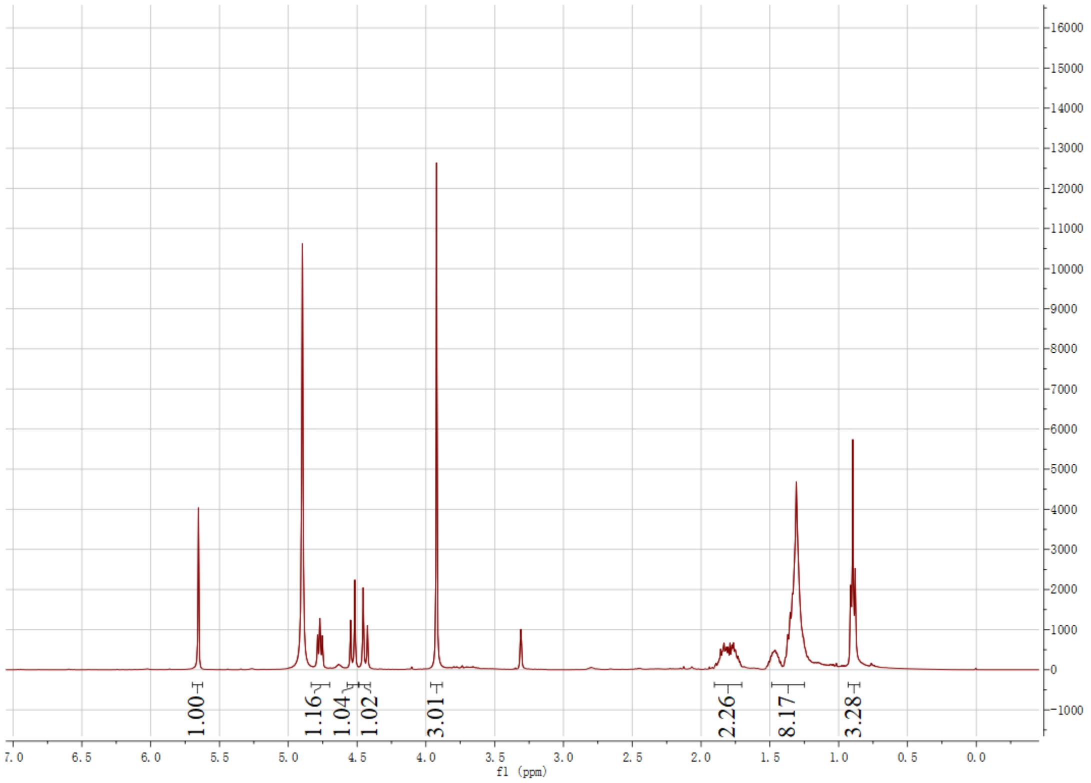

Figure S39 ^{13}C NMR spectrum of compound **6** (100 MHz, CD_3OD)

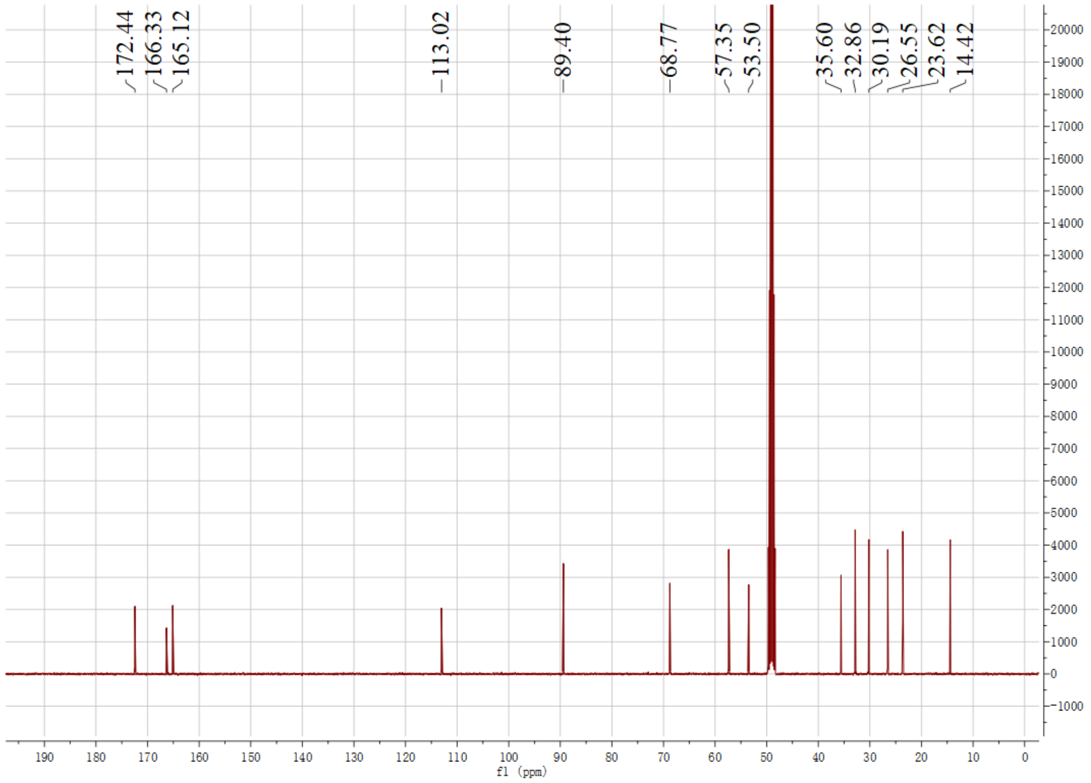

Figure S40 ^{1}H NMR spectrum of compound **7** (400 MHz, CD_3OD)

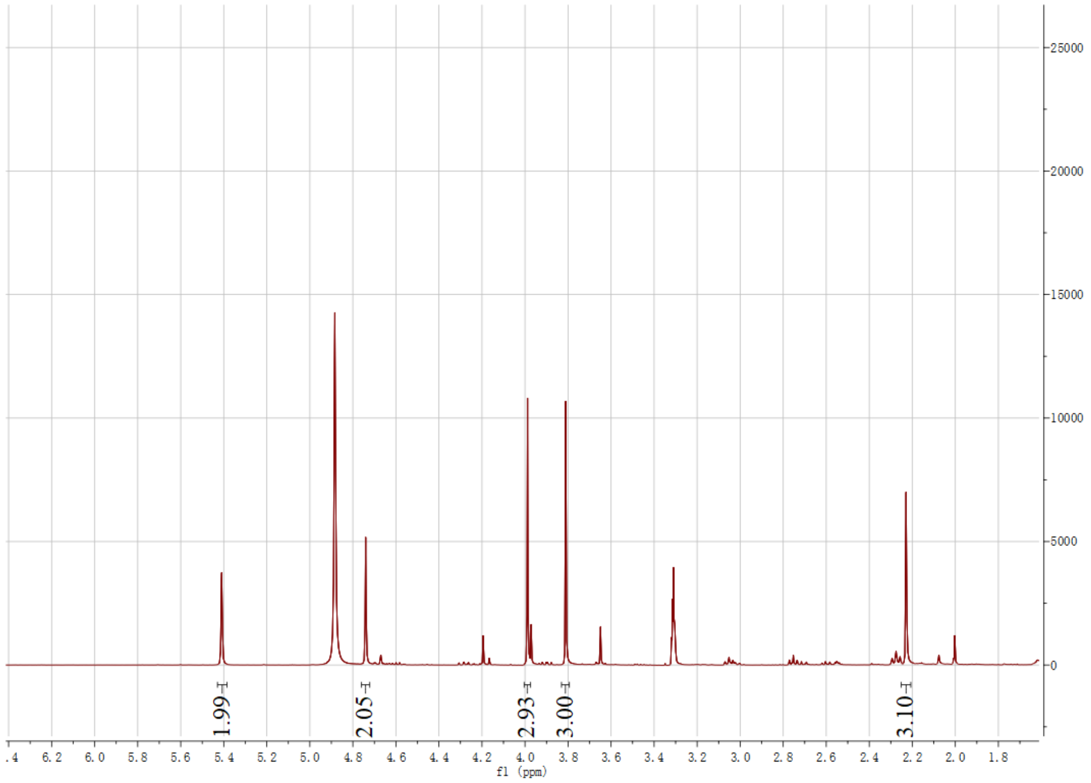

Figure S41 ^{13}C NMR spectrum of compound **7** (100 MHz, CD_3OD)

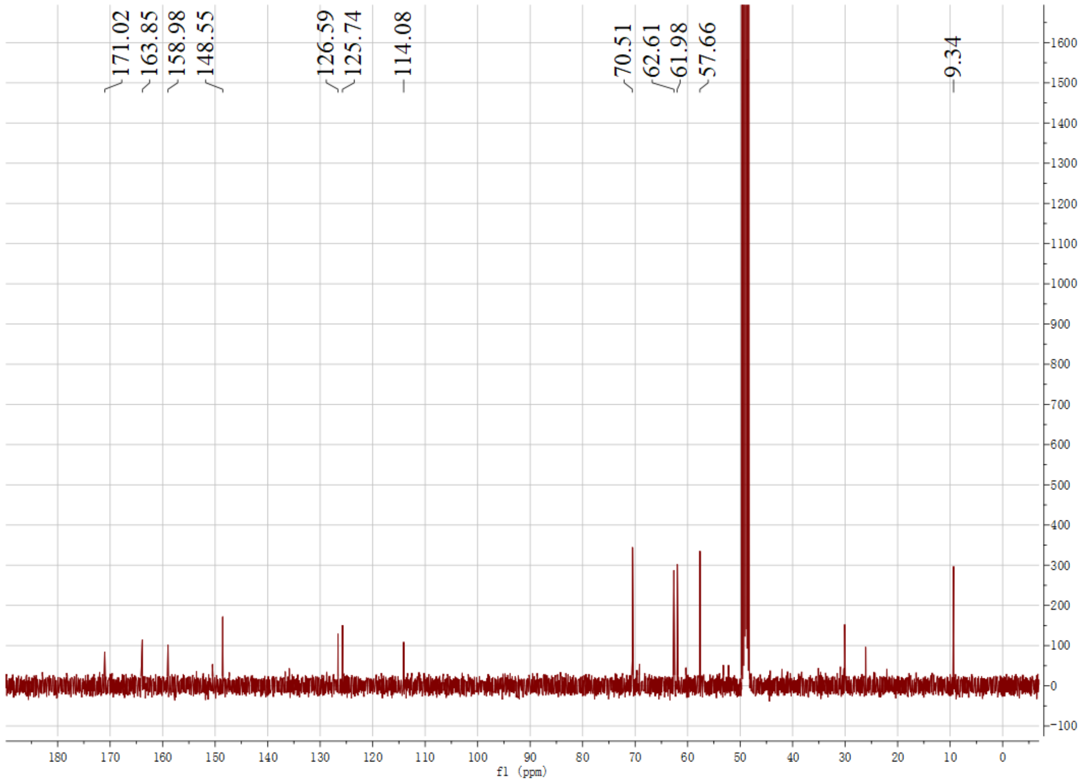

Figure S42 ^{1}H NMR spectrum of compound **8** (400 MHz, CD_3OD)

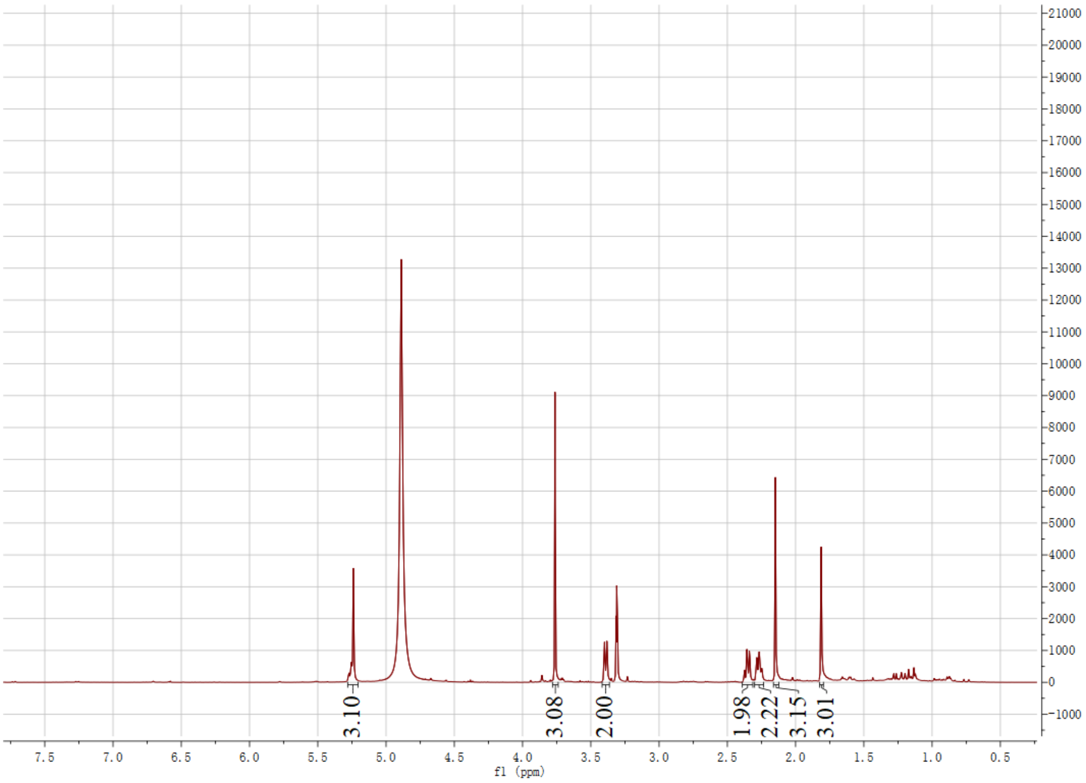

Figure S43 ^{13}C NMR spectrum of compound **8** (100 MHz, CD_3OD)

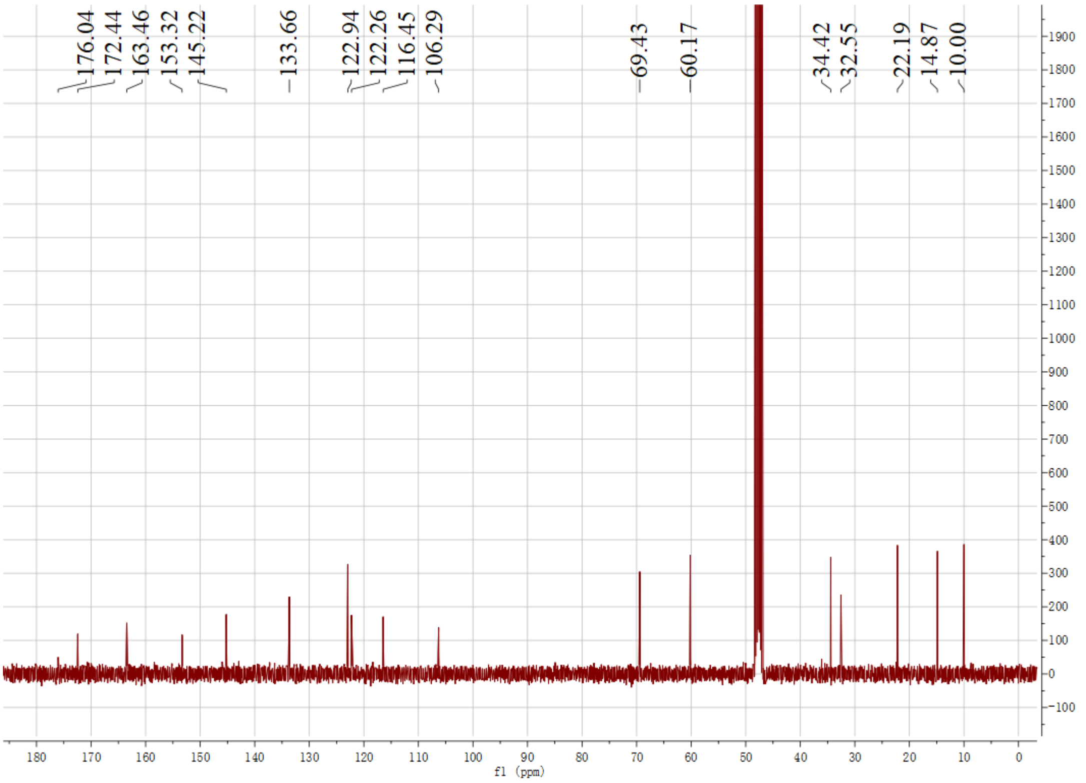

Figure S44 ^{1}H NMR spectrum of compound **9** (400 MHz, CD_3OD)

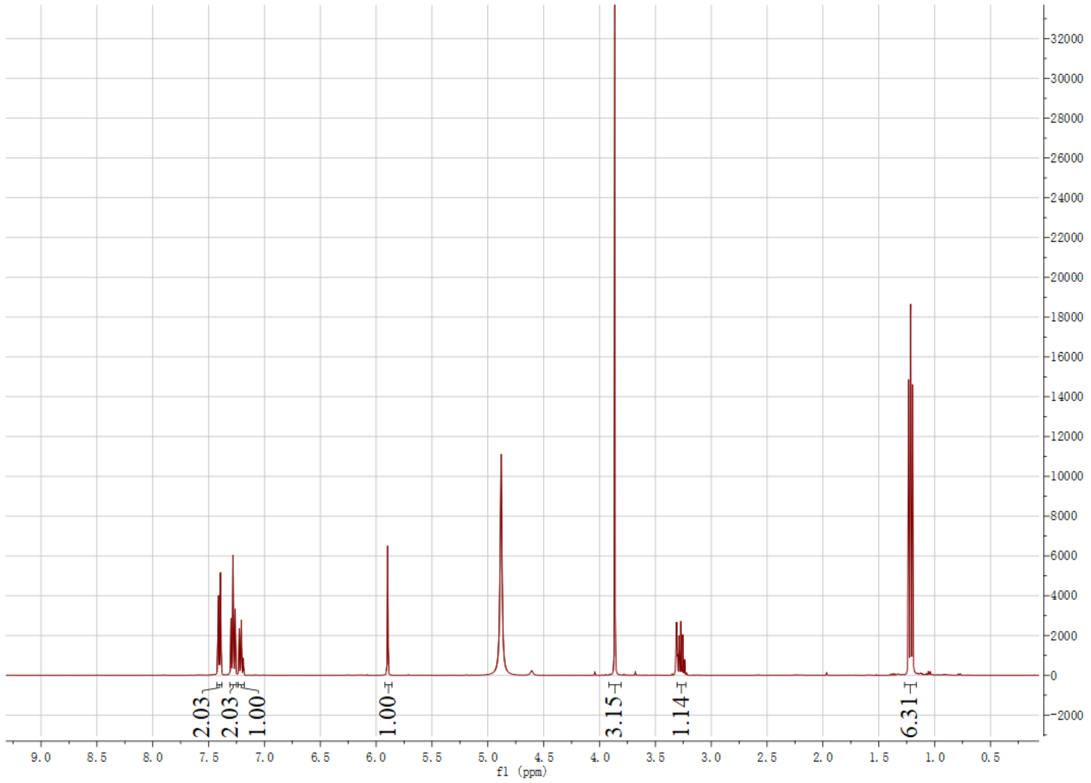

Figure S45 ^{13}C NMR spectrum of compound **9** (100 MHz, CD_3OD)

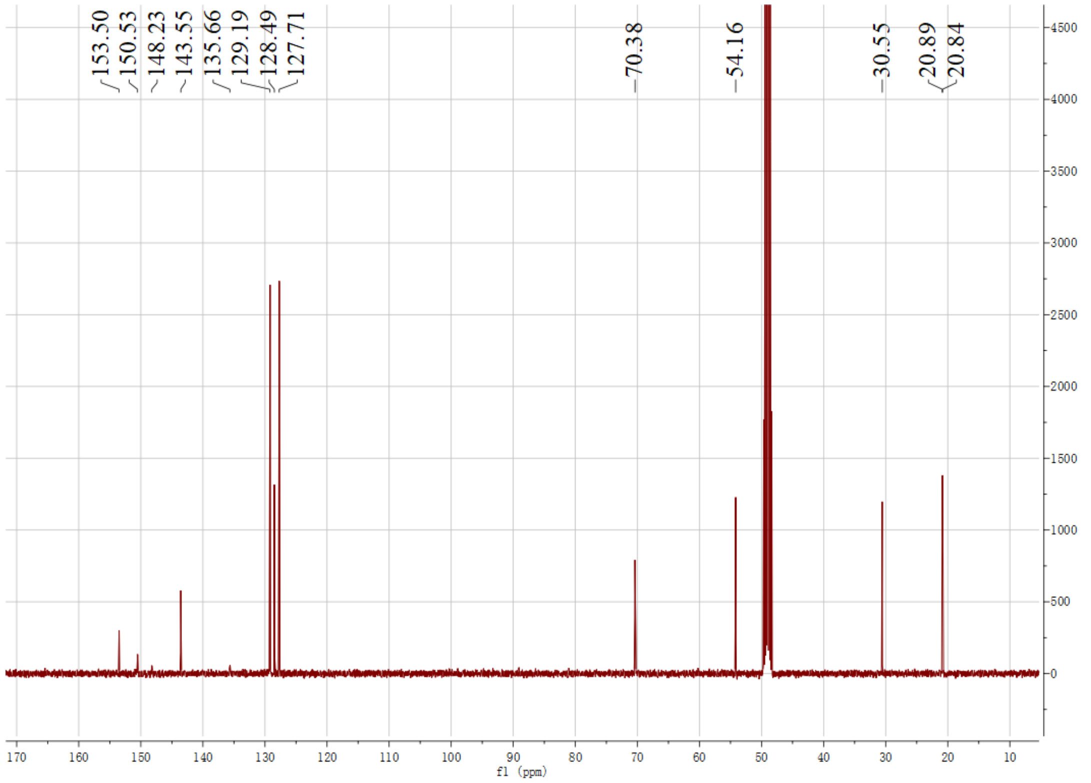

S1. Computational Section

Spartan 14 program (Wavefunction Inc., Irvine, CA, USA) was used for calculating Merck molecular force field (MMFF). Gaussian 16 program package1 was used for density functional theory (DFT) and time-dependent density functional theory (TDDFT) calculations. The conformational search was performed by a MMFF model, then the conformers with lower relative energies (< 10 kcal/mol) were subjected to geometry optimization with the DFT method at the B3LYP/6-31G* level. Vibrational frequency calculations were done at the same level to evaluate their relative thermal (ΔE) and free energies (ΔG) at 298.15 K. To obtain the energies of these low energy conformers, the geometry optimized conformers were further calculated at the B3LYP/6-311G* level was taken into consideration by using SMD.

Table S1. Energies of the dominative conformers of compounds **1-4**.

Compounds	No.	Structure	E (Hartree)	Population (%)
**1**	1	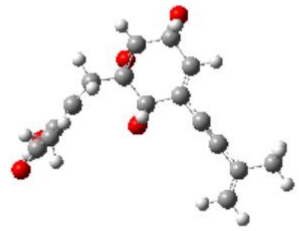	-996.59059095	10.47
	2	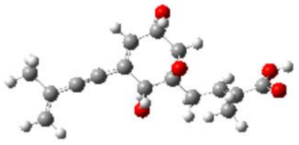	-996.59201459	38.35
	3	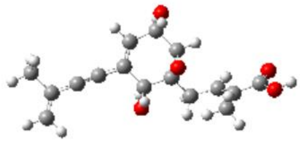	-996.59184472	42.95
	4	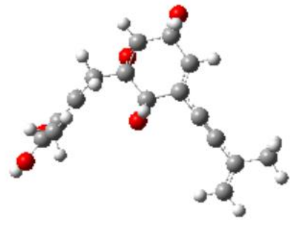	-996.59026377	8.23

**2**	1	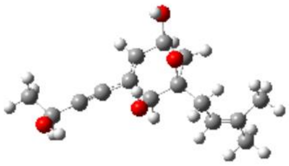	-884.40850353	100
	1	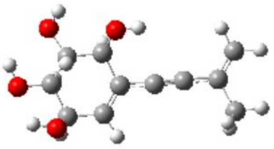	-728.38252251	42.97
**3**	2	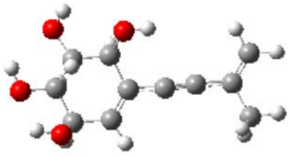	-728.38252245	42.97
	3	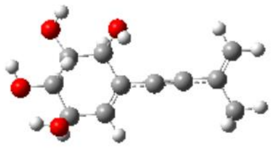	-728.38438755	14.07
**4**	1	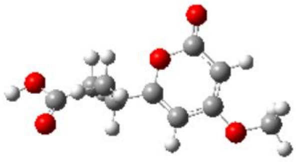	-764.43525102	52.86
	2	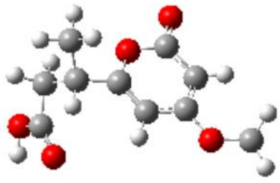	-764.43600676	47.14

Table S2. DP4+ analysis result table of model compounds **2a** and **2b** (experimental for **2**, isomer 1 for **2a**, and isomer 2 for **2b**).

Functional		Solvent?		Basis Set		Type of Data	
B3LYP		PCM		6-311+G(d, p)		Unscaled Shifts	
		DP4+	100.00%	0.00%	-	-	-
Nuclei	sp2?	Experimenta	Isomer 1	Isomer 2	Isomer 3	Isomer 4	Isomer 5
C	x	136.74	142.19	145.3			
C	x	134.18	139.71	139.19			
C	x	124.06	123.04	122.34			
C	x	118.95	118.55	118.61			
C		92.74	94.97	95.8			
C		82.27	82.56	82.79			
C		67.61	64.52	64.3			
C		66.1	64.46	63.54			
C		63.19	63.39	63.08			
C		60.34	59.89	59.6			
C		59	56.27	54.67			
C		32.2	29.61	27.12			
C		25.99	25.67	21.51			
C		24.62	19.22	16.95			
C		18.1	13.6	12.83			
